# Transposable Prophage Mu Is Organized as a Stable Chromosomal Domain of *E. coli*

**DOI:** 10.1371/journal.pgen.1003902

**Published:** 2013-11-07

**Authors:** Rudra P. Saha, Zheng Lou, Luke Meng, Rasika M. Harshey

**Affiliations:** Department of Molecular Biosciences & Institute of Cellular and Molecular Biology, University of Texas at Austin, Austin, Texas, United States of America; Institute of Molecular and Cell Biology (IMCB), A*STAR, Singapore

## Abstract

The *E. coli* chromosome is compacted by segregation into 400–500 supercoiled domains by both active and passive mechanisms, for example, transcription and DNA-protein association. We find that prophage Mu is organized as a stable domain bounded by the proximal location of Mu termini L and R, which are 37 kbp apart on the Mu genome. Formation/maintenance of the Mu ‘domain’ configuration, reported by Cre-*loxP* recombination and 3C (chromosome conformation capture), is dependent on a strong gyrase site (SGS) at the center of Mu, the Mu L end and MuB protein, and the *E. coli* nucleoid proteins IHF, Fis and HU. The Mu domain was observed at two different chromosomal locations tested. By contrast, prophage λ does not form an independent domain. The establishment/maintenance of the Mu domain was promoted by low-level transcription from two phage promoters, one of which was domain dependent. We propose that the domain confers transposition readiness to Mu by fostering topological requirements of the reaction and the proximity of Mu ends. The potential benefits to the host cell from a subset of proteins expressed by the prophage may in turn help its long-term stability.

## Author Summary

A majority of sequenced bacterial genomes harbor prophage sequences. Some prophages are viable, while others have decayed from accumulating mutations and genome rearrangements. Prophages, including defective ones, can contribute important biological properties such as antibiotic resistance, toxins, and serum resistance that increase the survival and ecological range of their hosts. We show in this study that the 37 kbp transposable prophage Mu exists in a unique configuration we call the ‘Mu domain’, where its two ends are paired, segregating the Mu sequences from those of the host chromosome. This is the largest stable chromosomal domain in *E. coli* mapped to date. The Mu domain configuration promotes low-level transcription from an early prophage promoter, which controls the expression of several genes, not all essential for phage growth. Some non-essential genes include DNA repair functions. We suggest that the Mu domain provides long-term survival benefits to both the prophage and the host: to the prophage in bestowing transposition-ready topological properties unique to the Mu reaction, and to the host in contributing extraneous DNA housekeeping functions.

## Introduction

Bacterial chromatin is spatially organized and condensed ∼1000 fold to fit inside a bacterial cell [1]. Referred to as a nucleoid, *E. coli* chromatin is organized in a series of negatively supercoiled loops [2], [3], segregated by dynamic domain barriers (defined as entities that prevent the free diffusion of supercoils) and compacted by several nucleoid-associated proteins (NAPs) including HU, IHF, Fis and H-NS [4], [5]. The chromosome is not randomly condensed, but rather has a ring organization with four structured macro-domains and two less-structured regions; interactions between these regions are highly restricted as determined by cytological and genetic analyses [6]. Macro-domains are thought to orchestrate chromosome movements during the cell cycle [7].

Supercoiling not only plays a vital role in compacting the chromosome, but a proper degree of supercoiling is crucial for all DNA-related processes [1]. Segregation of supercoils into topological domains protects these processes by preventing DNA breaks from relaxing the entire chromosome [2], [8]. The level of DNA superhelicity is tightly controlled by the combined activities of topoisomerases and histone-like proteins; the latter not only constrain negative supercoils and generate diffusion barriers for the formation of topological domains, but are also global regulators of gene transcription [4], [9]–[11]. The critical importance of DNA supercoiling in interconnecting chromosome structure and global gene transcription was reinforced in recent evolution experiments where supercoiling was observed to be under strong selection in *E. coli* populations [12].

Transposable phage Mu is a temperate phage that integrates into essentially random locations on the *E. coli* chromosome [13]–[15]. Transposition from mini-Mu plasmids *in vitro* requires DNA supercoiling for formation of a high-order transpososome within which the two Mu ends are interwound and synapsed [13], [16]. Supercoiling is inferred to be similarly important *in vivo* [17], where Mu end pairing during replicative transposition additionally requires a centrally located gyrase binding site SGS [18]. This site is the strongest such site studied, and is found only in Mu-like prophages [19]. Highly processive supercoiling by gyrase bound at the SGS has been proposed to propagate a supercoiled loop, with SGS at the apex, assisting transposase-mediated synapsis of Mu ends at the base [20].

*In vitro* studies found that the Mu transposase mediates an ordered interaction of three *cis*-acting sites - Mu L and R ends and an enhancer element E – which traps five supercoils before pairing the L and R ends in their reactive configuration [21]. The original goal of the present study was to test if the three sites interact in a similar order *in vivo* upon initiation of transposition. The *in vitro* studies employed Cre recombinase-mediated exchange at two strategically placed *loxP* sites to determine the topology of interactions between a given Mu site and the other two [16]. Using this same strategy *in vivo*, we found to our surprise that the ends were already paired in a prophage, and that the transposase was not essential for their pairing. We have investigated the basis of this pairing using both the Cre-*lox* system as well as the 3C crosslinking system [22], [23]. We show that Mu SGS is important for Mu end pairing, that other Mu *cis* and *trans* factors and several host NAPs contribute as well, and that the MuB protein, expressed at a low level in the prophage, likely provides a NAP-like function. We discuss the implications of this work for the maintenance of large selfish DNA elements on a bacterial genome.

## Results

### The two ends of the 37 kbp Mu prophage genome behave as if they are paired

The Cre-*loxP* site-specific recombination system has simple requirements, needing only two *loxP* sites; neither additional cofactors nor special DNA topology is required [24]. Cre recombinase can carry out both DNA inversion and deletion equally well, depending on the relative orientation of its *loxP* target sites [25]. The synapsis of *loxP* sites occurs by random collision, therefore the frequency of recombination between these sites will indicate their spatial proximity [26]. This property of Cre is used here to estimate the distance between recombining *loxP* sites engineered within the *E. coli* chromosome. Other site-specific recombinases have been similarly used in the past [6], [27].

**Figure 1:**
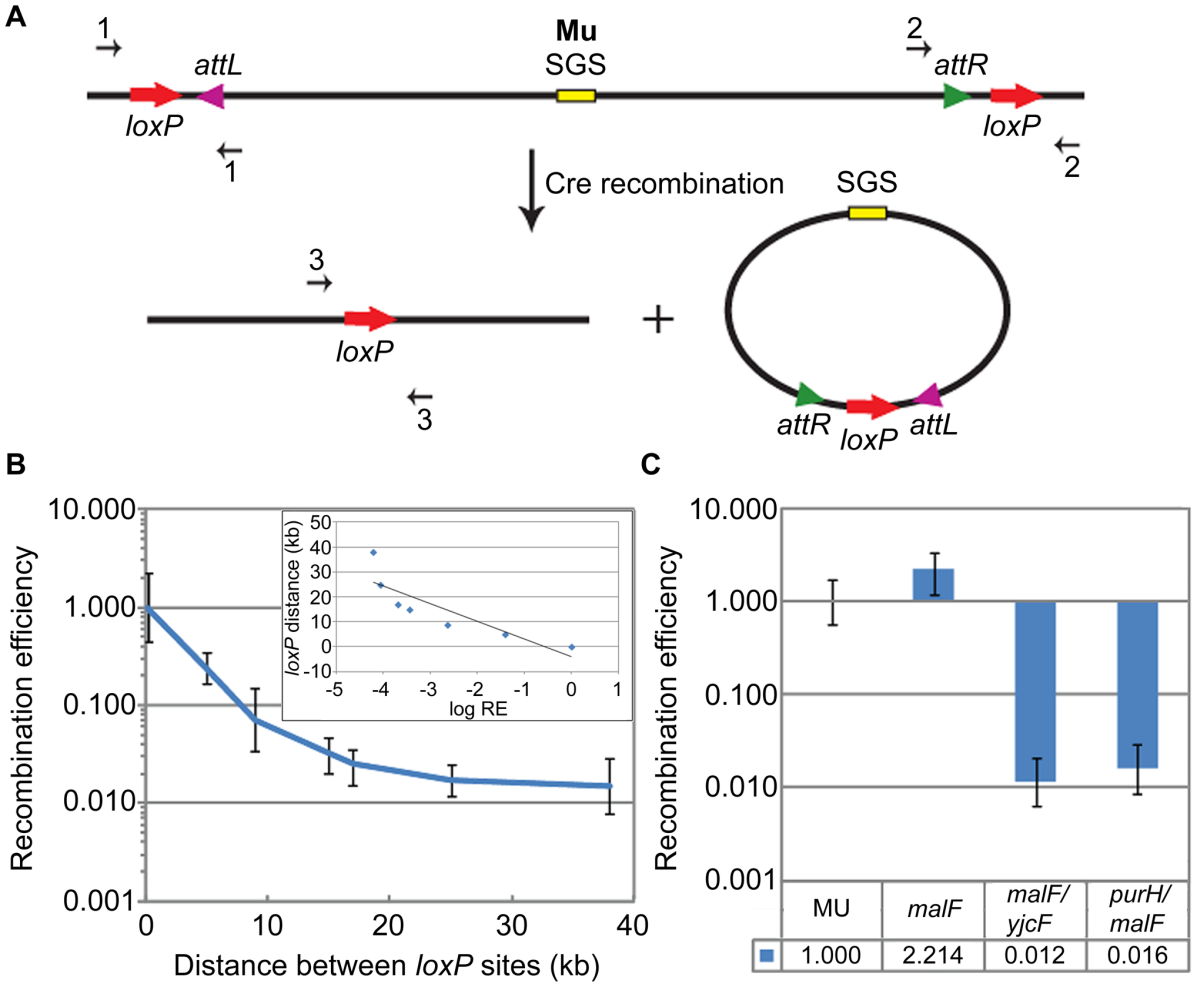
Cre recombination assay and *loxP* recombination as a function of distance. (**A**) Schematic shows directly oriented *loxP* sites flanking prophage Mu *attL* and *attR* (L/R in text), and the deletion products of Cre recombination. The excised Mu prophage will be lost during cell growth. Amounts of chromosomal starting substrate and product were assessed by qPCR across the *loxP* sites in the substrate (primer pairs 1 or 2) and product (primer pair 3). (**B**) RE of *loxP* sites as a function of distance on the *E. coli* chromosome (see Figure S2A). RE (recombination efficiency) calculation is described under Materials and Methods. RE of the 190 bp *loxP* pair was arbitrarily set at 1 (this pair is designated *malF* in Figure 1C). **Inset**: log (base e) RE values were plotted against *loxP* distance, and fitted to a straight line equation 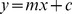, where 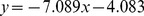; the slope (m) is ∼7 kbp. The plot is not quite linear because it includes two data points that fall within the plateau region of the graph (25 kbp and 37 kbp). A plot excluding the 37 kbp value is shown in Figure S2D. (**C**) RE of *loxP* sites placed ∼70 bp outside each end of the wild-type *malF*::Mu prophage in ZL524 (MU) is set at 1, and compared to the smallest (190 bp) (*malF*, ZL582) and largest (37 kbp) (*malF/yjcF*, ZL592 and *purH/malF*, ZL594) *loxP* pairs within the *malF* locus of the parent non-Mu strain (see Figure S2A). Bottom panel, precise fold differences in RE. Error bars are standard deviation from the mean.

The experimental strategy for assessing Cre recombination efficiency is diagrammed in Figure 1A, using as an example *loxP* sites flanking a Mu prophage. All assays in this study used the deletion reaction i.e. *loxP* pairs were configured in a direct orientation. Cre recombinase was provided from a plasmid, and reaction conditions optimized as described under Materials and Methods (Figure S1). After recombination, the intervening DNA between the *loxP* sites will be excised, leaving one *loxP* site on the chromosome and the other site in the excised product; the latter will be lost during cell growth. As diagrammed in Figure 1A, the amounts of substrate and product, both chromosomal, were assessed by qPCR after amplification across the *loxP* sites with appropriate primers. Recombination efficiency (RE) was calculated as the ratio of the recombination product to the starting substrate as described in Materials and Methods.

The distance-dependence of *loxP* recombination on the *E. coli* chromosome was first assessed by varying the distance between a pair of directly oriented *loxP* sites from ∼190 bp to 37 kbp engineered within the *malF* locus (Figure S2A). This locus was chosen because the Mu prophage we wished to monitor in later experiments was located there. In the *loxP*-engineered strains, the log of *loxP* RE decreased linearly over distance, giving a first order decay function (Figure 1B and inset; see also Figure S2D). *loxP* sites were next engineered on either side of a *malF*::Mu lysogen, ∼70 bp outside each L and R end as shown in Figure 1A (Figure 1C, this wild-type *loxP*-Mu-*loxP* construct in ZL524 is labeled MU throughout). The RE of the MU *loxP* sites (set at 1) was closest to the *loxP*-site pair placed 190 bp apart within *malF* in the non-Mu strain (Figure 1C, *malF*). To control for recombination at distances similar to the length of the Mu prophage around this region of the chromosome, *loxP* sites were placed 37 kbp upstream (*yjcF*) or downstream (*purH*) of a *loxP* site in *malF* in the non-Mu strain (see Figure S2A). The RE of both these *loxP* pairs was similar (Figure 1C), and reflected their linear distance as determined from the graph in Figure 1B. We conclude that reduction of the linear 37 kbp distance between the L and R Mu ends to a distance equivalent to 190 bp as measured by Cre recombination, is indicative of some form of ‘synapsis’ of the Mu ends.

### The centrally located strong-gyrase-site (SGS) within prophage Mu is important for end-synapsis

**Figure 2:**
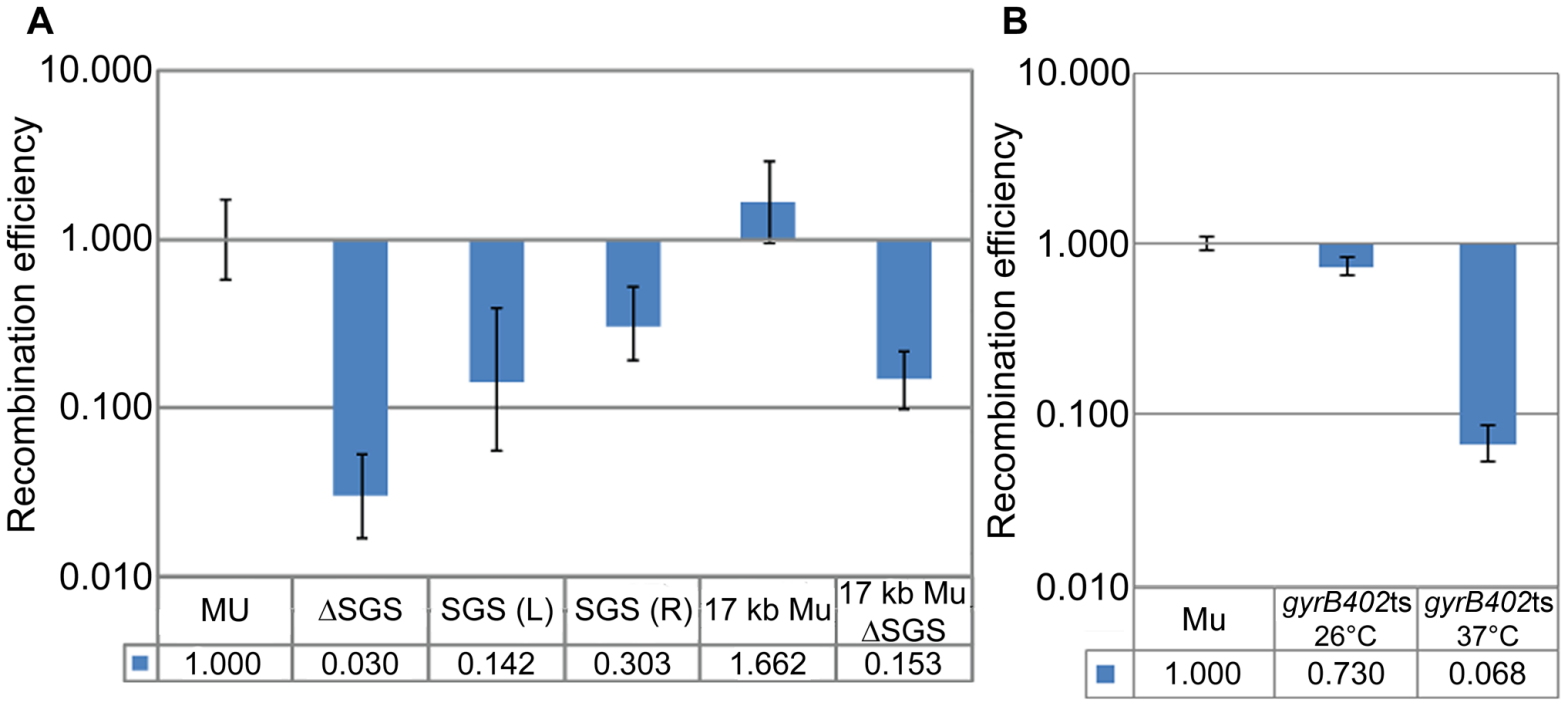
Importance of SGS and DNA supercoiling to Mu end synapsis. (**A**) RE of *loxP* sites flanking Mu in SGS-deleted, SGS-displaced to left (L) or right (R) of center, and reduced-Mu genome strains. MU (ZL524), ΔSGS (ZL562), SGS(L) (ZL573), SGS(R) (ZL578), 17 kb Mu (RS020), 17 kb ΔSGS (RS025). (**B**) RE of *loxP* sites flanking a Mu*c*+ lysogen (ZL911) and its isogenic *gyrB*ts strain (ZL941) measured at 26°C and 37°C, using the rhamnose-inducible Cre plasmid as described under Materials and Methods. Other descriptions as in Figure 1.

Central location of SGS (Figure 1A) is obligatory for optimal replication of Mu after prophage induction [18]. Deletion of this site results in inefficient pairing of Mu ends *in vivo*, as judged by a low efficiency of transposase-mediated 3′ nicking at the ends [28], [29]. Pato and colleagues have proposed that the requirement for SGS *in vivo* but not *in vitro* (where the distance between the Mu ends is typically ∼2 kbp on mini-Mu plasmid substrates), is an adaptation for aiding synapsis of reactive sites located at large distances [20]. We therefore tested the importance of both the presence and position of SGS on the RE of *loxP* sites flanking Mu. Deletion of the central SGS decreased *loxP* recombination 30-fold, while an asymmetric location of SGS to the left (L) or right (R) of center in separate strains showed 7- and 3- fold reduction, respectively, compared to wild-type (Figure 2A; the RE values of 0.142±0.086 and 0.303±0.11 for the SGS (L) and SGS (R) prophage strains are not significantly different at p<0.01). Reduction of the Mu genome by 20 kbp upon introducing symmetrical 10 kbp deletions on either side of SGS (i.e. genome size of 17 kbp), still showed Mu end synapsis on the smaller Mu genome, which was disrupted by SGS deletion (Figure 2A). A similar reduction of effective distance was not observed when an SGS site was engineered at the center of *E. coli* DNA segments ranging from 5–37 kbp, each flanked by *loxP* (Figure S2B, C). This shows that the SGS site alone is not sufficient to synapse distant *loxP* sites; Mu sequences are required in addition.

Because the effect of SGS is proposed to be mediated via gyrase-promoted supercoiling, the temperature sensitive gyrase allele *gyrB402*ts was introduced into a Mu*c* ^+^ lysogen, which is not temperature inducible. Since gyrase temperature-sensitive mutants are reported to have decreased supercoiling even at the permissive temperature [30], *loxP* REs were measured at both permissive (26°C) and non-permissive (37°C) temperatures. *loxP* sites behaved as if they were unpaired only at the non-permissive temperature (Figure 2B; the wild-type *loxP*-Mu*c* ^+^-*loxP* construct is labeled Mu).

We conclude that DNA supercoiling is important for reducing the distance between the Mu ends, that SGS plays a critical role in this process when located centrally either on a 37 kbp or a 17 kbp Mu genome, but that SGS does not similarly contribute when located within non-Mu *E. coli* DNA. These results support the Mu end-pairing function of SGS as deduced by the transposition/replication results of Pato and colleagues. However, our data were derived in the absence of prophage induction i.e. presumably in the absence of the transpososome proposed to stabilize the synapsed ends.

### SGS-mediated Mu DNA synapsis does not extend far outside the Mu ends

**Figure 3:**
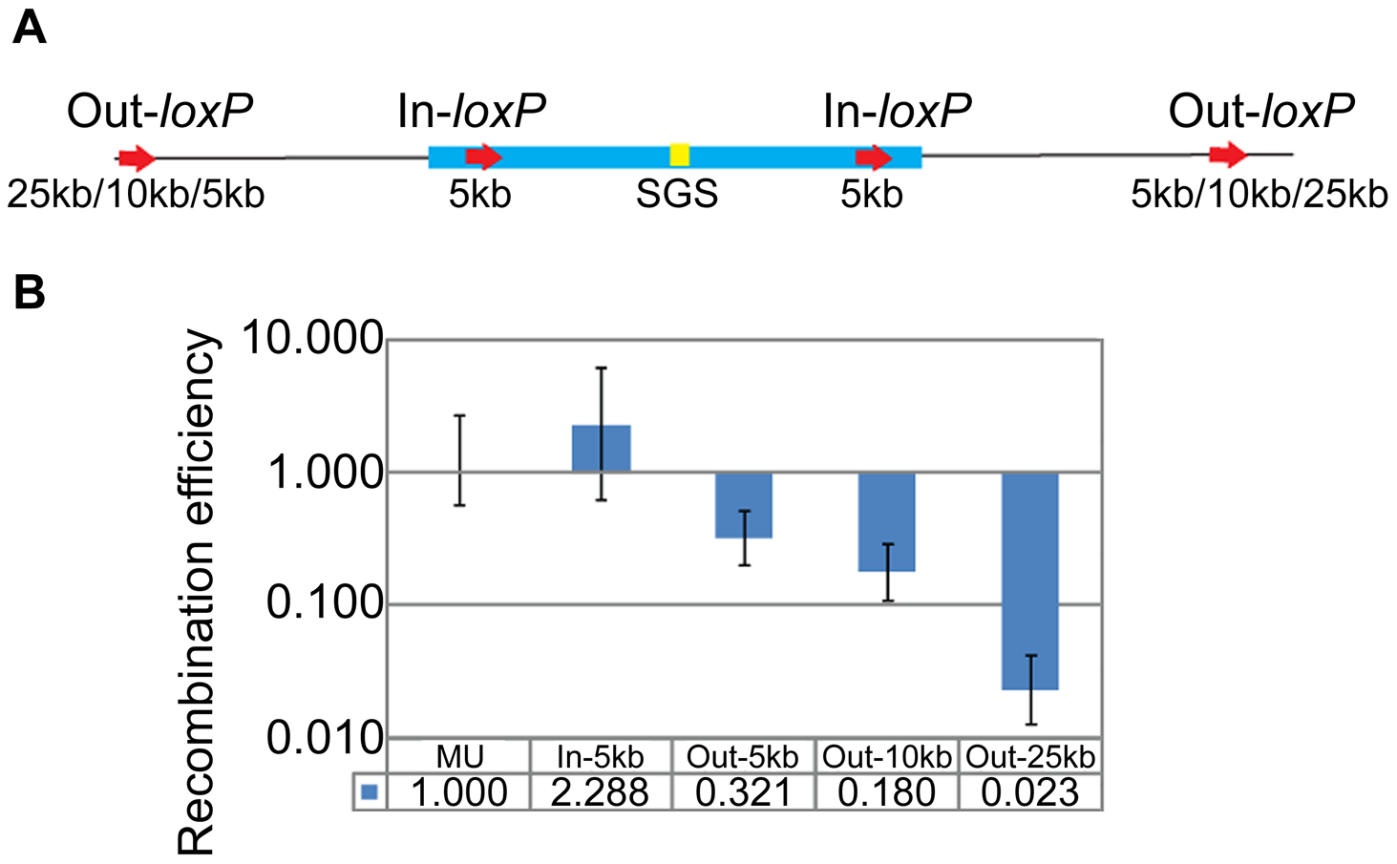
Propagation of the Mu domain outside Mu ends. (**A**) Position of *loxP*-site pairs inside (In) or outside (Out) Mu. The exact location of the sites is given in Table S1. (**B**) RE of *loxP*-site pairs diagrammed in A. In-5 kb, Out-5 kb etc. refers to RE of pairs of symmetrically placed sites within and outside the L and R ends of Mu in different strains at the indicated distances. MU (ZL524), In-5 kb (ZL732), Out-5 kb (ZL720), Out-10 kb (ZL724), Out-25 kb (ZL728).

To test whether Mu ends define the base of the SGS-mediated Mu DNA synapsis loop, we moved the *loxP*-site pairs symmetrically from 5 kbp inside Mu (In-*loxP*) to 5–25 kbp outside Mu (Out-*loxP*) in separate strains (Figure 3A). RE of the internal *loxP*s (In-5 kbp) was similar to wild-type MU, while that of external *loxP*s [Out-5 kbp (0.321±0.12) and Out-10 kbp (0.18±0.07)] decreased 3–5 fold over 5 and 10 kbp distances, which is a total linear distance of 47 and 57 kbp between the *loxP* pairs, respectively (Figure 3B). Synapsis was no longer evident between the Out-25 kbp pair (87 kbp linear distance), as determined by >40 fold lower RE values (0.023±0.011) compared to wild-type MU. We conclude that the SGS effect extends 5–10 kbp outside Mu ends into the flanking *E. coli* DNA; beyond this length, the RE is reflective of the linear rather than paired distance between the *loxP* sites (see standard graph in Figure 1B).

### 3C reveals an interaction between Mu prophage ends

In 3C analysis, protein-protein and protein-DNA crosslinking by formaldehyde is used to permanently capture interactions between two genomic loci [22]. After appropriate restriction enzyme digestion and ligation at low DNA concentration, the suspected junctions can be probed by PCR using locus-specific primers. While this methodology has been widely used to generate DNA contact maps in eukaryotic cells [31], it is only beginning to be used in bacteria [23], [32]. We applied this strategy to test the proximity of Mu L and R ends predicted from the Cre-*loxP* recombination assay.

**Figure 4:**
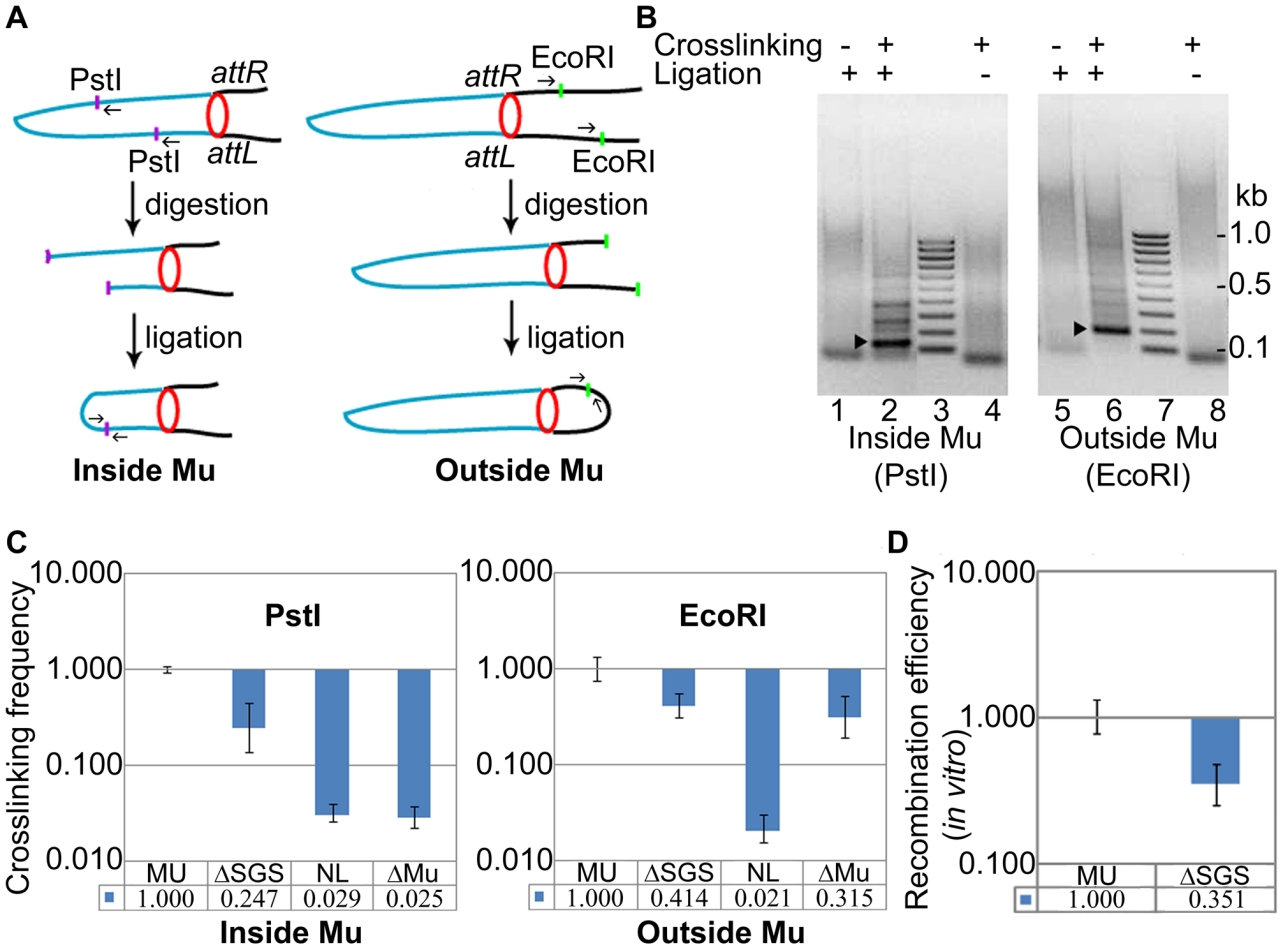
Interaction of prophage Mu ends probed by 3C methodology. (**A**) Experimental design (see text and Materials and Methods). Blue line, Mu DNA; black line, *E. coli* DNA; purple and green dots, PstI and EcoRI sites, respectively; small arrows, primers used to amplify the DNA ligation product; red circle, paired L and R ends. (**B**) PCR products of ligation. Left: Primers were designed to produce a 155 bp fragment after PstI digestion-ligation (lane 2, arrowhead), and a 195 bp fragment after EcoRI digestion-ligation (lane 6, arrowhead); the fainter bands above the specific products in lanes 2 and 6 could not be re-amplified, hence are non-specific. The specific products were not observed in uncrosslinked (lanes 1, 5) and unligated (lanes 4, 8) samples. The band migrating at ∼100 bp in these lanes is non-specific. Lane 3, 7 DNA size marker ladder. (**C**) Quantitation of the ligation products. The qPCR signal obtained from wild-type MU ligation was set at 1. Crosslinking efficiency is defined as the ratio of qPCR signal from the ligation product in the ΔSGS strain compared to that in its wild-type parent. NL is the signal obtained from the non-ligated, crosslinked product in the wild-type reactions shown in lanes 4 and 8, and ΔMu is a similar control in a strain where Mu has been excised from ZL524 via recombination of the flanking *loxP* sites. The same set of primer pairs were used for all strains in either the PstI or the EcoRI panels. MU (ZL524), ΔSGS (ZL562), ΔMu (ZL580). (**D**) *In vitro* Cre-*loxP* recombination of the cross-linked DNA from the indicated strains before digestion with restriction enzymes.

To assay DNA interactions both inside and outside Mu, we used digestion at PstI and EcoRI sites, respectively, whose positions in *malF*::Mu are shown in Figure 4A. The PstI sites closest to the L and R ends inside the Mu genome are ∼27 kbp apart, whereas the closest EcoRI sites outside the Mu genome are ∼52 kbp apart (L-proximal site is ∼13 kbp upstream and R-proximal site is ∼2 kbp downstream of the prophage location). Products of the expected size were detected for both PstI and EcoRI joints (Figure 4B, arrowheads) in a crosslinking-ligation dependent manner. Their identities were confirmed by DNA sequencing. No PCR product corresponding to the joining of DNA cut at two EcoRI sites within Mu was detected, possibly due to cross-linked proteins interfering with ligation.

The ligation products obtained in Figure 4B were quantified further by qPCR and compared to similar reaction products from an isogenic ΔSGS strain (Figure 4C). Crosslinking efficiency is defined as the ratio of qPCR signal from the products of the ΔSGS strain compared to those from its wild-type parent. Cross-linking efficiency of PstI ends within Mu was 4-fold (0.247±0.11) higher in the presence of SGS compared to its absence (Figure 4C, left), and that of EcoRI ends outside Mu was 2.5 fold (0.414±0.106) higher under similar conditions (Figure 4C, right). The fold-differences in crosslinking efficiencies versus recombination efficiencies, of wild-type MU and its ΔSGS derivative (Figure 4C vs Figure 2A), are likely due to differences in methodology. Background (non-specific) levels of signal generated by unligated but crosslinked samples (lanes 4 and 8 in Figure 4B) are shown in each panel (NL), along with similar controls for DNA around the *malF* locus in a strain where Mu had been excised (Figure 4C, ΔMu).

For additional and independent quantitation of the crosslinked product, we subjected it to Cre-*loxP* recombination *in vitro* using a titrated amount of Cre. While the *in vitro* efficiency cannot be directly compared to the *in vivo* efficiency, at the lowest Cre concentration required to observe ∼30% recombination in the wild-type crosslinked substrate, the RE of *loxP* sites in the ΔSGS substrate was 3-fold (0.351±0.11) lower than the wild-type (Figure 4D). Taken together, these results are an independent confirmation of the close spatial proximity of Mu ends in the Mu prophage and the important contribution of SGS to this arrangement. We shall henceforth refer to this apparent Mu-loop as a ‘Mu domain’.

### Importance of *cis-* and *trans*-acting Mu transposition factors to the Mu domain

**Figure 5:**
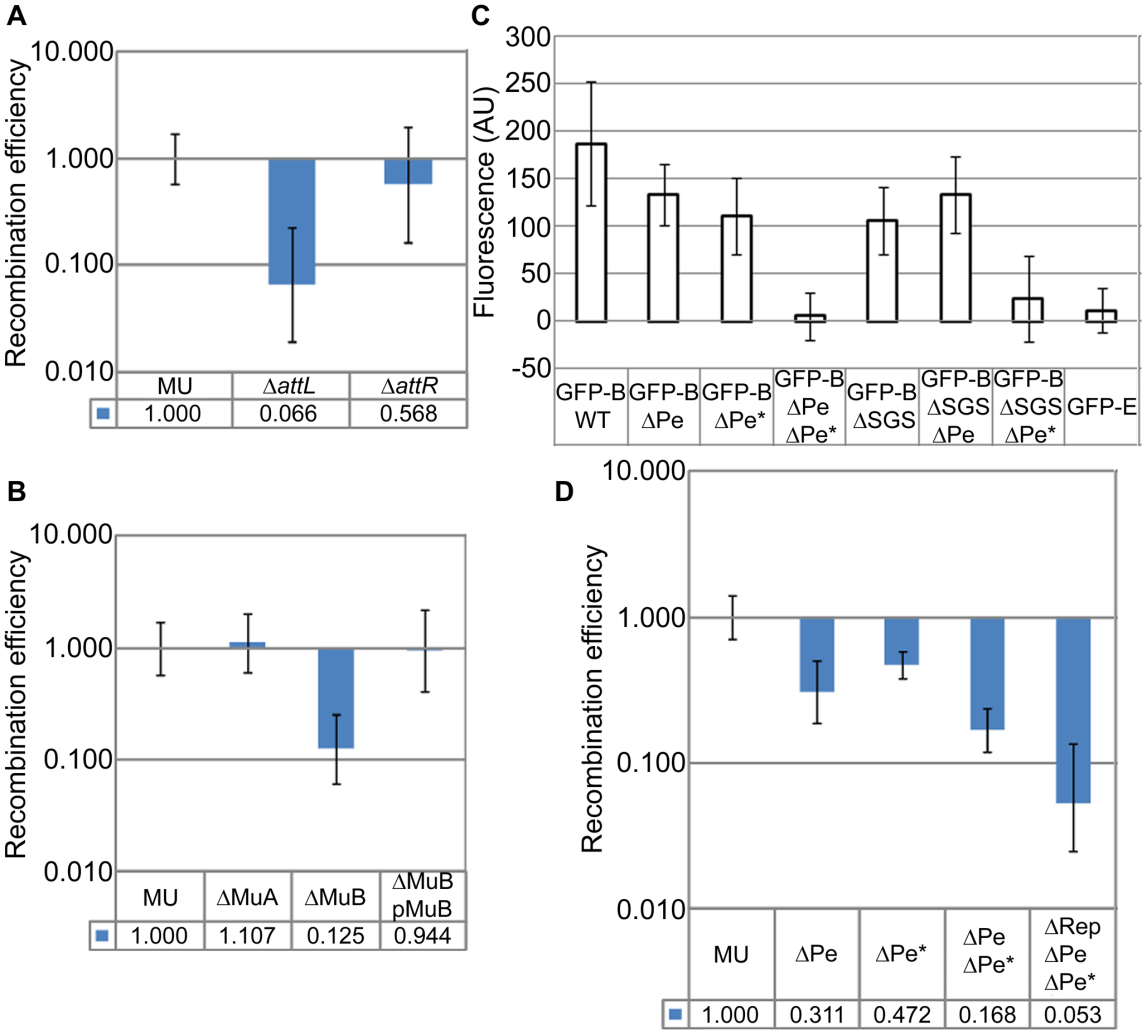
*Cis-* and *trans*-acting Mu transposition factors required for Mu domain formation, and discovery of early rightward transcripts in the prophage. (**A**) RE of *loxP*s in Δ*attL* (ZL552) and Δ*attR* (ZL556) strains compared to their wild-type parent MU. (**B**) RE of *loxP*s in strains deleted for Mu*A* (ZL536) and Mu*B* (ZL530) genes, and MuΔ*B* strain complemented with MuB from plasmid pJG8 (pMuB). (**C**) Expression of EGFP-MuB in a wild-type (WT) Mu lysogen and isogenic strains carrying SGS and early promoter deletions. Excitation and emission wavelengths were 488 nm and 507 nm, respectively. Fluorescence values were subtracted from the background fluorescence of the parental Mu strain without the EGFP-MuB (MP1999), and expressed as arbitrary units (AU). Error bars are standard deviation from the mean. Strains: Wild-type Mu*c*ts prophage expressing EGFP-MuB (RS033) and its deletion derivatives ΔPe (RS048), ΔPe* (RS102), ΔPe ΔPe* (RS103), ΔSGS (RS088), ΔSGS ΔPe (RS106), ΔSGS ΔPe* (RS107) and EGFP-MuE (RS101). (**D**) RE of *loxP*s in Mu prophage strains carrying early promoter deletions. ΔPe (RS053), ΔPe* (RS092), ΔPe ΔPe* (RS093), ΔRep ΔPe ΔPe* (ZL951). In panels A, B and D, MU is ZL524.

Given that the SGS effect is Mu-specific, we wondered if the L and R ends of Mu are important for closing the Mu loop at its base, and if so, whether the Mu transposase (A protein), which binds to the Mu ends, is expressed in the prophage. We therefore individually deleted the L and R ends as well as the Mu*A* and Mu*B* genes from the prophage (MuB regulates MuA function allosterically; [13]). Deletion of the L end showed a 15-fold (0.066±0.049) reduction of RE, but deletion of the R end had no significant effect (Figure 5A). Deletion of the Mu*A* gene had no effect, but deletion of Mu*B* had an 8-fold effect (0.125±0.064) (Figure 5B). To confirm the *B* gene deletion result, MuB was supplied to this strain from a plasmid [pMuB (pJG8; [15])]; RE levels were restored to wild-type in this strain. The non-requirement for the R end and for MuA, but the requirement for MuB, in Mu domain formation/maintenance, will be discussed later.

Mu*A* and *B* genes are expressed from the early lytic Pe promoter, expected to be repressed in a prophage (Figure S3A; [33]). To determine if MuB was expressed in the lysogen, we engineered into the prophage genome a functional EGFP-MuB fusion [34]. Low-level expression of MuB was detected in all the cells in this strain in the absence of Mu induction (Figure S3B). 5′-RACE-PCR experiments showed transcripts originating from Pe, as well as from a second site internal to Mu*A*, which we have named Pe* (Figure S3C). Pe* has significant homology to the sigma 70 sequence (Figure S3D). Deletion of each of these promoters had a small effect on EGFP-MuB expression as measured by fluorescence, but deletion of both promoters eliminated expression (Figure 5C). Note that either promoter deletion will render the strain uninducible for Mu lytic growth. A control EGFP fusion to a late Mu gene *E* expressed during lytic growth, showed no fluorescence in the prophage (Figure 5C). Thus, the transcription results cannot be due to spontaneous Mu induction in a subpopulation of cells, because all cells expressed EGFP-MuB, none expressed EGFP-MuE, the Pe deletion interrupts the lytic growth program, and the Pe* deletion does the same by disrupting the Mu*A* gene.

To test if the low-level expression of MuB from Pe and Pe* was related to the Mu domain, we monitored EGFP-MuB expression in a ΔSGS strain. Deletion of SGS diminished EGFP-MuB fluorescence compared to its wild-type parent. To test if this reduction was specific to either Pe or Pe* we measured MuB-EGFP fluorescence in the ΔSGS strain carrying separate Pe or Pe* deletions (Figure 5C). Expression from Pe was the most impacted by deletion of SGS.

Single or double deletion of Pe and Pe* led to a reduction in the RE of *loxP* sites flanking the wild-type Mu strain, with the double-promoter deletion showing a value similar to that of Mu*B* gene deletion (Figure 5D, compare with Figure 5B). As described above, the Pe-Pe* deletion disrupts the Mu*A* gene as well as early transcription, thereby eliminating Mu lytic development. This allowed us to delete the Mu *c* gene encoding the lysogenic repressor Rep in the Pe-Pe* deletion strain, in order to test its contribution to the Mu domain (see Figure S3A). The data showed that absence of Rep caused an additional 3-fold decrease in RE (0.053±0.028) (Figure 5D).

We conclude that the L end is important but that the R end is dispensable to the Mu domain. MuB, but not MuA, is important for domain formation/maintenance, and Rep likely contributes as well. Low levels of MuB are expressed in the lysogen from both Pe and a newly identified promoter Pe*. The Mu domain configuration is important for transcription from Pe but not Pe*. Thus activity of Pe is domain-dependent, while that of Pe* is not.

### Cellular NAPs are critical for maintenance of the Mu domain

**Figure 6:**
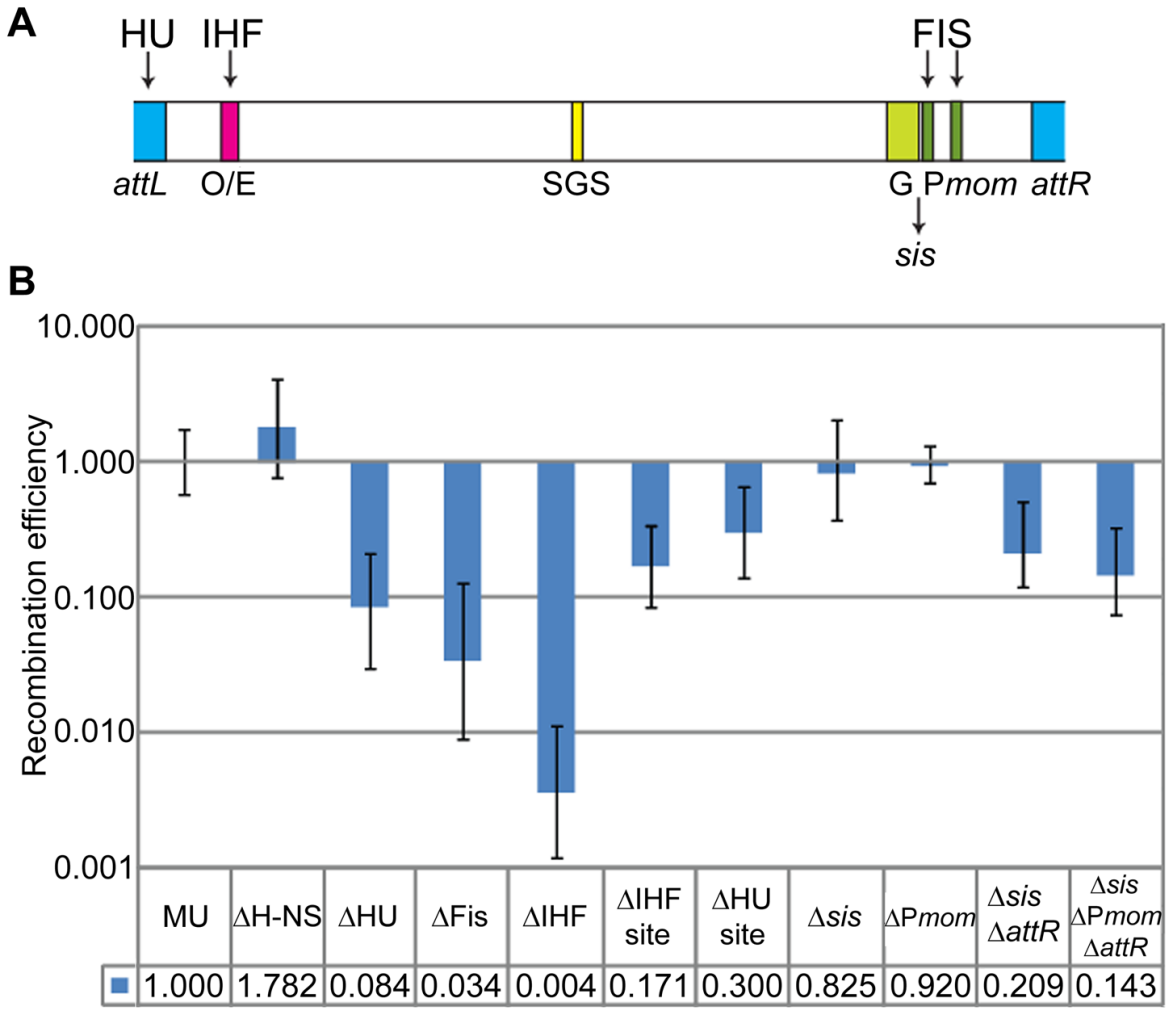
Role of *E. coli* NAPs and their binding sites on the Mu genome, in Mu domain configuration. (**A**) Schematic showing binding sites for IHF, Fis and HU on the Mu genome. O/E, operator/enhancer; G, invertible G segment; *sis*, Fis-binding enhancer site for G inversion; P*mom*, promoter for the *mom* gene. (**B**) RE of *loxP* sites in strains either deleted for the indicated NAPs or for their binding sites on Mu. Strains: MU (ZL524), ΔH-NS (ZL624), ΔHU (ZL634), ΔFis (ZL614), ΔIHF (ZL604), ΔIHF site (ZL656), ΔHU site (ZL652), Δ*sis* (ZL660), ΔP*mom* (ZL670), Δ*sis* Δ*attR* (ZL662), Δ*sis* ΔP*mom* Δ*attR* (ZL672).

*E. coli* NAPs such as H-NS, IHF, FIS, and HU are implicated in maintaining chromosomal supercoiled domains via their DNA bending and bridging properties. In contrast to the major *E. coli* NAPs, which were found largely scattered throughout the nucleoid, H-NS was reported to form two compact clusters per chromosome, sequestering and juxtaposing into these clusters numerous H-NS regulated DNA segments distributed throughout the chromosome; deleting H-NS led to substantial chromosome reorganization [23]. IHF, FIS and HU have in addition, specific binding sites on the Mu genome, from where they exert effects on Pe transcription, G-segment recombination, and transposition [13] (Figure 6A).

To determine the importance of these NAPs to the Mu domain, we assayed for changes in RE of *loxP* sites flanking Mu in strains individually deleted for genes expressing these proteins (Figure 6B). Absence of H-NS had no effect on the Mu domain. Absence of HU and of Fis had 12 to 30-fold effects, respectively (0.084±0.054 and 0.034±0.025). The strongest effect was observed in the absence of IHF, which essentially abrogated the Mu domain. That NAP deletions do not affect Cre recombination *per se* was controlled for by simultaneously monitoring the recombination of *loxP* site pairs placed outside the Mu domain in both wild-type and ΔNAP strains (see Materials and Methods).

To test if the effects of IHF, Fis and HU were exerted at the specific binding sites for these proteins on the Mu genome, we deleted these sites individually within the prophage. The dramatic reduction in RE seen in the IHF mutant was not observed with deletion of the IHF binding site (Figure 6B). However, the 6-fold effect observed (0.17±0.086), could be due to the negative effect on Pe transcription from deleting the IHF site [35]–[38]. Deletion of the HU-binding site at the Mu L end had a small effect, while deletion of the Fis-binding enhancer site *sis* (Δ*sis*) had no effect. Recent experiments have identified a set of three Fis-binding sites within the promoter region of the *mom* gene near the R end [39]. A deletion spanning all three sites (ΔP*mom*) also had no effect. However, a combination of *sis-attR* or *sis*-P*mom-attR* deletions reduced RE 5–7 fold (0.209±0.091 and 0.143±0.068). We conclude that IHF, HU and Fis affect the Mu domain configuration primarily via their global effects on chromosome structure.

### The domain organization is unique to Mu, and is not observed for prophage λ

**Figure 7:**
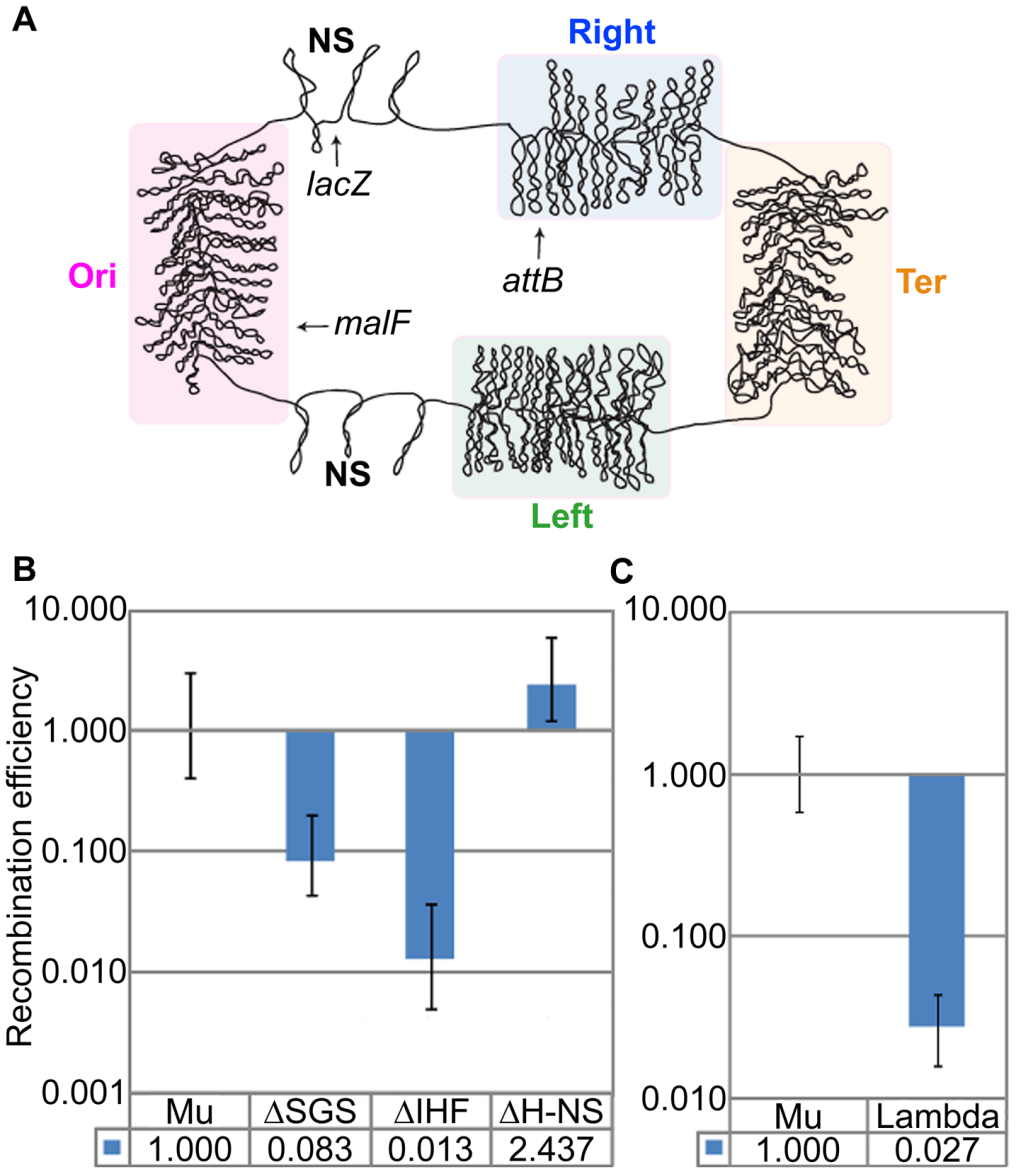
Mu domain at an NS chromosomal location, and probing for a domain configuration for prophage λ. (**A**) *E. coli* chromosome macro-domains and position of prophages at the loci examined in this study. (**B**) RE of *loxP* sites flanking Mu*c*+ prophage (Mu) located in *lacZ*, and its isogenic mutants. Mu (ZL911), ΔSGS (ZL921), ΔH-NS (ZL931), ΔIHF (ZL936). (**C**) RE of *loxP* sites flanking prophage λ (ZL808) compared to Mu (ZL911).

The four structured macro-domain regions of the *E. coli* chromosome are called Ori, Right, Ter, and Left; the two less or non-structured regions (NS) are located on either side of Ori [7] (Figure 7A). The *malF*::Mu prophage used in all the experiments thus far is located in the Ori macro-domain. To determine if the Mu domain organization is specific to its location in a macro-domain, we also tested for its presence in a *lacZ*::Mu*c* ^+^ prophage located in the less-structured NS region between Ori and Right. The results were similar to those seen with the *malF*::Mu prophage, with similar negative effects of deletion of SGS or absence of IHF on the domain structure (Figure 7B, compare to similar data in Figures 2A and 6B). Absence of H-NS had no effect at this location as well.

If the domain organization of Mu were designed to pre-engage Mu ends in a transposition-ready mode for lytic growth, might a similar arrangement be expected for other prophages that depend on pairing of their ends at the start of lytic growth? Prophage λ is an example of an insertion element which must pair its *attL* and *attR* ends for excisive recombination from the *E. coli* chromosome [40]. The *attB* insertion site of λ is in the Right macro-domain (Figure 7A). To determine if λ prophage ends were paired, *loxP* sites were engineered outside the 48.5 kbp λ genome, as done for Mu. In contrast to Mu, the Cre RE of these sites reflected the linear distance between the λ ends (Figure 7B). Thus, domain organization does not occur for phage λ, and may be specific for Mu.

## Discussion

The 37 kbp Mu prophage domain we report in this study, inferred from Cre recombination and supported independently by a crosslinking assay, is the largest stable chromosomal domain in *E. coli* mapped to date. ‘Stable’ implies that the configuration is long-lived enough to be consistently detected by both genetic recombination and biochemical crosslinking. This sets the Mu domain apart from the dynamic configuration of the 400–500 supercoiled domains that condense the *E. coli* nucleoid [4]. While the nucleoid exhibits different degrees of compaction around the circular genome referred to as macro-domains [41], [42], and distant chromosomal regions within these macro-domains cluster via H-NS [23], the specific supercoiled domain adopted by the Mu prophage is unique in that it represents a more or less permanent feature of the *E. coli* chromosome. The formation/maintenance of the Mu domain requires contributions from both prophage and its host, as discussed below.

### Cre-*loxP* recombination as a reporter for chromosomal domains

The organization of the bacterial chromosome into supercoiled domains has been studied earlier using different strategies: trimethylpsoralen binding, electron microscopy, transcription of supercoiling sensitive genes, or site-specific recombination by Res and Int [2], [3], [43], [44]. The Res system initially yielded an average domain size of 25 kbp for a non-essential region spanning ∼100 kbp of the *Salmonella typhimurium* genome [43]. Recombination efficiency was found to decrease linearly with the distance between target *res* sites over the entire region analyzed. Since the topological constrains of the resolvase reaction requires the *res* sites to be housed within the same supercoiled domain [45], it was concluded that topological domains are dynamic, with stochastically distributed end points. Using an improved system in which the Res protein was designed to have a shorter half-life, the average domain size in *Salmonella* was re-calculated to be approximately 10 kbp [27]. A domain size of 10 kbp agrees with results obtained in *E. coli*, where measurements from electron microscopy and the spread of DNA relaxation from double strand breaks were used to estimate a domain size centered around 10 kbp, within a 2–66 kbp range [3]. When prophage λ *attL* and *attR* sites, which can recombine within an Int synapse arranged by random collision [40], [46], were placed all over the chromosome, Int recombination efficiencies suggested that accessibility of individual loci was not uniform in different regions of the *E. coli* and *Salmonella* chromosomes [6], [44]. The macro-domain organization of the *E. coli* chromosome deduced from these and cytological studies is shown in Figure 7A [6].

The utility of the Cre-*loxP* system in probing chromosomal domains stems from the simple requirements of Cre recombination, the ease of integrating *loxP* sites at desired chromosomal locales, and the *in vivo* distance-dependence of the reaction revealed in this study. The ∼7 kbp value of the slope derived for Cre recombination (Figure 1B, inset), which is the distance at which there is a 50% probability that barriers to supercoil diffusion exist, defines a domain size in this region of the *E. coli* chromosome in reasonable agreement with the average 10 kbp domain estimate of Postow et al. [3]. Since the Mu genome is much larger than the average *E. coli* chromosomal domain, efficient Cre recombination at *loxP* sites placed at the extremities of Mu would be consistent with these sites being contained within a domain. We note that although *loxP* recombination by Cre is analogous to *attL*-*attR* recombination by λ Int in not requiring negative supercoiling and in following the random collision mechanism, the distance-dependence of recombination frequencies observed for the two systems *in vivo* cannot be strictly compared because of many differences in experimental conditions such as bidirectional (Cre) versus unidirectional (λ Int) configurations of the recombining sites, differences in recombinase levels and reaction times, different methods for estimating REs (colony color, PCR, qPCR), and differences in growth media and growth conditions [6], [44].

### Sequestration of Mu into an independent supercoiled domain: Mu and host factors

The domain organization of prophage Mu, anchored by Mu L and R ends, was observed at two structurally different regions of the *E. coli* chromosome (Figures 1–4, and Figure 7). The pairing of the neighboring DNA arms was seen to extend 5–10 kbp outside Mu into the *E. coli* DNA (Figure 3). Both SGS and gyrase were critical to domain integrity (Figure 2). The requirement of SGS for the formation of the prophage domain is consistent with the role for SGS originally proposed in promoting Mu end synapsis for transposition [18], [47]. In each case, the gyrase-mediated processive supercoiling initiated at the center of Mu may be cemented at the L and R ends by either the transpososome or by boundary proteins and the Mu repressor (see below) to establish two functionally distinct DNA domains.

The Mu L end and the MuB protein, but not the R end and the MuA transposase, were required for formation of the prophage domain (Figure 5A, B). The non-requirement of the R end is puzzling. While the L and R ends have specific binding sites for MuA and for lysogenic repressor Rep [48], they also have an AT-rich character [49]. We speculate that while the R end normally *is* required, other AT-rich elements can substitute in its absence. This possibility is suggested by the observation that an effect of R-end deletion on the Mu domain is only manifested when combined with deletion of AT-rich Fis-binding sites near this end (Figure 6B). In the *E. coli* genome, AT-rich elements or A-tracts are over-represented and distributed ‘quasi-regularly’ with a 10–12 bp periodicity throughout the genome, organized in ∼100 bp long clusters [50]. Such elements have been proposed to constitute a ‘structural code’ for DNA compaction via NAP binding. Thus, in the absence of the R end, synapsis of Mu termini could be assisted by NAPs. Similarly, absence of the MuA transposase could be compensated for by the presence of the lysogenic repressor Rep, which shares sequence homology with the transposase and binds to Mu ends [48], [51], [52] (Figure 5D).

The requirement for MuB in domain organization/stability is consistent with the detectable but low level domain-dependent transcription from the early lytic promoter Pe and activity of a domain-independent promoter Pe* in the prophage state (Figure S3 and 5C). Deletion of the IHF site is expected to impact Pe transcription (Figure 6). How might MuB assist the Mu domain? MuB is known to have a binding preference for AT-rich sequences [53], [54] and can compete with NAPs for such sequences [15]. The distribution of EGFP-MuB fluorescence throughout the cell is noteworthy in this context (Figure S3B). A potential prophage-specific NAP-like activity would be a novel role to be identified for this multifunctional protein, which affects target site selection, modulates transposase activity and promotes target site immunity during transposition [13]. We note that an earlier Res recombination study in *Salmonella* using a *B*am Mu prophage, did not detect the Mu domain we report here [55]. It is likely that the lack of MuB impacted the results, including the possibility that the *B*am Mu prophage used in that study carried in addition a large insertion of an Amp-Lac segment near the R end that might have disrupted the symmetry of the SGS site; additionally, *Salmonella* and *E. coli* chromosomes show differences in supercoiling [56].

The most abundant NAPs in *E. coli* - Fis, HU, H-NS and IHF - engender at least partially overlapping functions, as absence of any one of these proteins results in rather subtle phenotypes [4], [57]. The formation of a stable Mu domain, however, was essentially abrogated by the absence of IHF, and was strongly impeded by the absence of Fis or HU; lack of H-NS had no effect on domain establishment (Figure 6). Although IHF regulates MuA and MuB levels from Pe [35]–[38], and Fis has been implicated in regulating Rep levels [58], [59], while HU is required for Mu transposition [60], [61], the magnitude of the effects of deleting the genes for these proteins was far greater than deleting their known binding sites. Thus, IHF, HU and Fis proteins appear to facilitate Mu domain formation independently of their site-specific interactions within the Mu genome relevant to transposition or to inversion of the G-segment via site-specific recombination [13]. Rather, the process is likely assisted by their global role in nucleoid organization.

### A model for the Mu prophage domain: Functional implications

**Figure 8:**
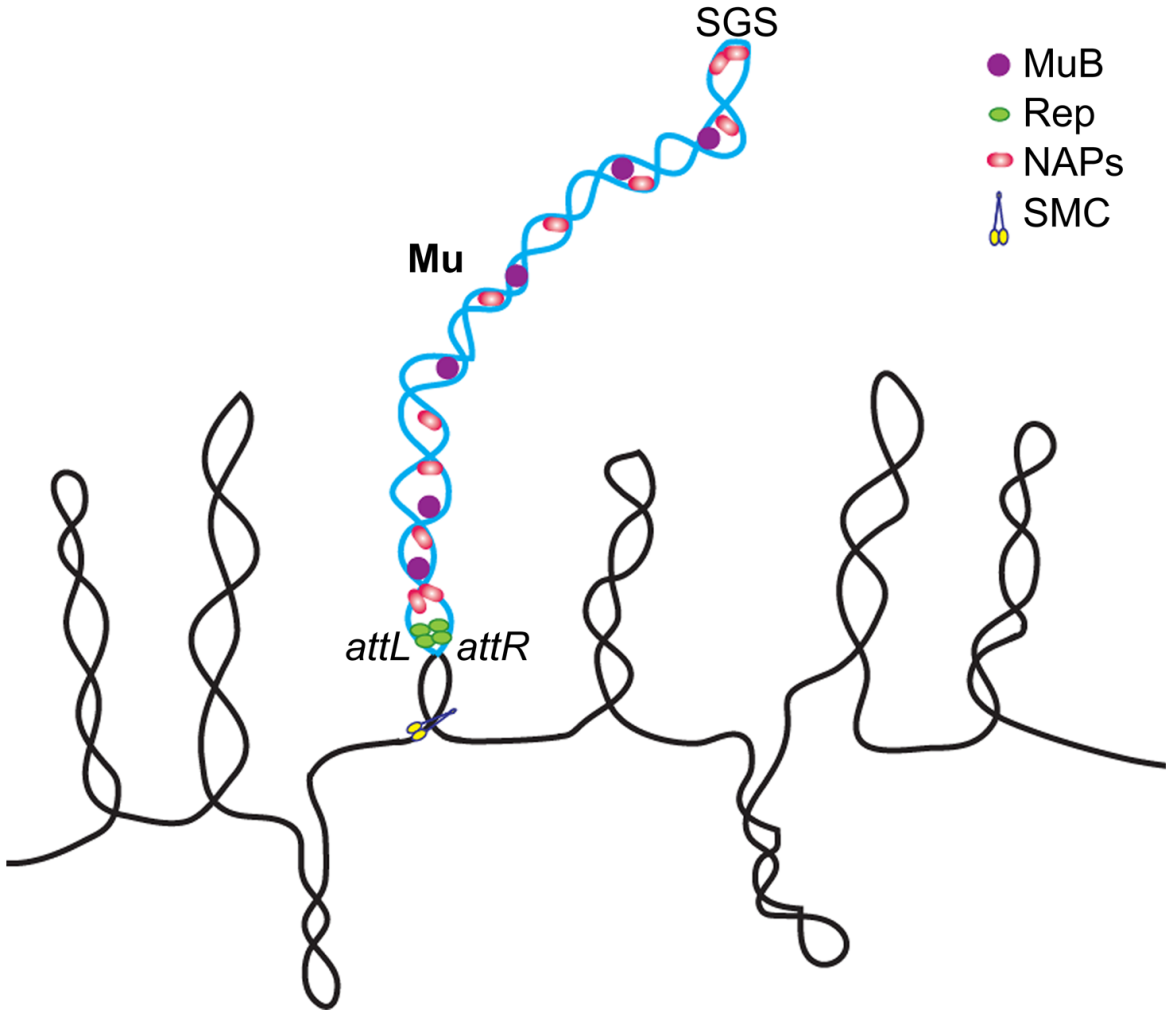
Model of the Mu prophage domain. See text for description. The single supercoiled Mu loop shown is not intended to imply absence of branching. NAPs not tested in this study, such as SMC-like proteins, may also be involved in domain maintenance. A variation of this model was proposed earlier for replicating Mu, to account for strong MuB binding only within Mu [47].

The model for the Mu prophage domain that we propose (Figure 8) incorporates our present findings with the earlier proposal of Pato and colleagues [20]. According to this model, the processivity of gyrase bound to the SGS site located at the center of the Mu genome, helps to align the left and right arms and promote synapsis of the L and R termini. End-binding proteins such as the transposase had been proposed earlier to seal the Mu loop and stabilize the synapse. In the prophage state, it is likely that the Mu repressor Rep rather than MuA is involved in end pairing, since more repressor molecules are expected to be present. This would explain why deletion of Mu*A* had no effect on the Mu domain (Figure 5B). However, Rep has a lower affinity for the ends compared to the MuA [48]. Since MuA must be expressed at a low level from the Pe transcript, a scenario where both proteins contribute to closing the Mu loop is also a plausible mechanism for ensuring domain stability. MuB likely serves as a NAP [15], assisting cellular NAPs such as FIS and IHF in stabilizing the Mu domain. Other essential NAPs such as SMC proteins could also be involved in maintaining the Mu domain [62] (Figure 8). The domain configuration promotes basal level transcription from Pe, ensuring domain maintenance via MuB.

Like Mu, prophage λ depends on pairing of its *attL* and *attR* termini for excision prior to entry into lytic growth. Yet a domain organization was not detected for λ (Figure 7). The contrasts between Mu and λ in the architecture of their prophage genomes perhaps reflect the topological and mechanistic distinctions of the transposition and recombination reactions, respectively, which set forth each phage on the lytic path. A closed supercoiled domain may not offer a special advantage to λ excision as the Int bound *attL* and *attR* sites find each other by random collision, even when present on unlinked DNA molecules [46]. In contrast, the Mu synapse is arranged by an ordered series of interactions between three sites – the L/R ends and the enhancer E – all of which must be present in *cis*, on the same DNA molecule [13]. The first Mu interactions between E and R, which subsequently engage L to generate an LER synapse, trap five DNA supercoils within the synapse [63]. The organization of this highly specific topological filter, that presages the chemical steps of Mu transposition, would be aided by the SGS-assisted formation of a self-contained supercoiled domain [16], [64].

Prophages, despite being largely repressed in gene activity, are major contributors of genome diversity in some bacterial species [65], [66]. Many of these prophages appear to be in a state of mutational decay. Both intact and defective prophages can contribute important biological properties to their bacterial hosts. The best example of ‘fixation’ of defective prophage genes are Shiga toxin genes in *Shigella dysenteriae*, *sopE2* in *Salmonella enterica*, *sspH* and pertussis-like toxin genes in *Salmonella typhi* [66]. Some of the glycosyl transferase (*gtr*) genes of *Salmonella* may be another such example [67]. The genes for virulence factors and antibiotic resistance often carried by prophages, add to the fitness of their hosts, thereby ensuring long-term self-propagation as well [68]. In prophage λ, a small subset of its genes - *rex*, *lom* and *bor* - transcribed at a low level in the prophage, are involved in conferring on the host bacterium resistance to lytic phages or to serum [69], [70]. In this context, the low level transcription we observe from Pe may also confer some advantage to the host (Figures 5C and S3B, C). This transcript encompasses a number of ‘semi-essential’ (SE) genes whose functions are largely unknown (Figure S3A). A subset of such genes could potentially benefit the host. The *gem* gene in the SE region has been reported to modulate host ligase and gyrase functions even in a lysogen [71], and *gam* encodes an orthologue of the eukaryotic protein Ku, which participates in double-strand break repair [72]. Pe*, whose activity is domain independent, could serve as a back-up promoter for the expression of Mu*B* and other SE genes during brief periods when the Mu domain is disrupted, either stochastically or for functional reasons such as chromosome replication. We suggest that the positioning of SGS within the Mu genome and the SGS-induced structuring of the Mu domain are beneficial to both the phage and the host, in keeping the former transposition ready and in providing the latter the NAP-like MuB protein and proteins such as *gem* and *gam* that function in cellular physiology. The proposal that the Mu prophage confers a fitness advantage is supported by earlier competition assays under glucose-limiting conditions, that demonstrated a selective advantage for Mu and other prophage containing bacteria over their prophage-free counterparts [73].

### Summary

This is the first report of a distinct organization of a prophage genome into a stable supercoiled ‘loop’ structure which we call the Mu domain. A closed-loop structure of the Mu genome, where synapsis of prophage ends is assisted by processive supercoiling by gyrase bound to a centrally located SGS site, had been proposed earlier to aid the transposition of Mu during its lytic phase of growth. The novel result we report is that the Mu domain exists even in the quiescent prophage state, and requires in addition to SGS, several Mu proteins and host NAPs for its formation/maintenance. The Mu domain regulates an early promoter that controls the expression of several genes, many with unknown functions; known functions include DNA transposition, DNA repair and nucleoid structure maintenance. A domain structure likely benefits the prophage by holding its ends in a transposition-ready configuration, and benefits the host by providing extra housekeeping functions. The latter proposition can be tested in long-term evolution experiments, where a Mu lysogen with an intact SGS site would be expected to outcompete one without an SGS site.

## Materials and Methods

### Strain construction

All strains used in this work were derivatives of *E. coli* K-12 and are listed in Table 1. Plasmids are listed in Table 2. Gene disruptions, substitutions, deletions and insertions on the chromosome were made using the phage λ red-mediated homologous recombination methodology [74], [75]. Position of mutations is listed in Table S1. Primer sequences are listed in Table S2. In strains with the temperature-inducible Mu*c*ts prophage, all incubation steps were at 30°C. All gene deletions with the kanamycin gene *kan* replaced the start codon to the stop codon of the gene to be deleted by amplifying the flanking regions of the gene using primers with 50 nt homology extensions using pKD4 as the *kan* template, and selecting for kanamycin resistance (50 µg/ml). For Mu *A*, *B* and *c* gene deletions, the 1.6 kbp *kan* cassette was retained at the site of deletion in order to maintain symmetry of the SGS site. Other deletions were created by a two-step procedure: First, the DNA to be deleted was replaced by a dual selection cassette - either *cat-sacB* (amplified from strain SIMD30; [76]) or *kan-ccdB* (amplified from pKD45, where the *ccdB* gene is under a rhamnose-inducible promoter; [77]) into the sequence to be replaced. Selection for the cassettes was on chloramphenicol (Cam) (100 µg/ml) or kanamycin. Next, the cassettes were replaced by homologous recombination with appropriate DNA to create the desired mutation, selecting on either LB plates supplemented with 6% sucrose or 0.5% rhamnose to eliminate *cat-sacB* or *kan-ccdB*, respectively. The gyrase mutant in the Mu*c* ^+^ lysogen was made by moving the *gyrB402*ts allele [78] flanked by a *cat-sacB* cassette from ZL940 into ZL911 by P1 transduction, selecting for Cam^R^. *loxP* sites flanking λ prophage were created by inserting them on either side of the λ integration site in *attB* site prior to lysogenization with λcI857 (Cam) obtained from SIMD30. All constructs were confirmed by DNA sequencing. In the ΔHU strain, *hupA* is deleted [79]. In the ΔIHF strain, *himA* is deleted [80].

**Table 1:** Strains.

Strains	Genotype*	Source (ref.)
AB1157	*thr-1, araC14, leuB6, lacY1, tsx-33, qsr'-0, glnV44, galK2, LAM-, Rac-0, hisG4, rfbC1, mgl-51, rpoS396, rpsL31(strR), kdgK51, xylA5, mtl-1, argE3, thi-1*	M. Pato
MP1999	AB1157, *recB*, *recC*, *sbcB*, *malF*::Mu*c*ts62	M. Pato
MG1655	F^−^ λ^−^ *ilvG* ^−^ *rfb*-50 *rph*-1	Lab stock
EC1512	*argE thi ilv gyrB402*	Filutowicz (1983)
SIMD30	W3110 *lacDU169 galKTYR145UAG* (λc1857Δ*cro-bioA*) *[l int'::cat-sacB]*	Datta (2008)
ZL524	MP1999, *loxP* sites inserted 61 bp and 70 bp outside *attL* and *attR*, respectively	This study
ZL530	ZL524, Mu*c*ts *B*::*kan*	This study
ZL536	ZL524, Mu*c*ts *A*::*kan*	This study
ZL552	ZL524, Mu*c*ts Δ*attL*	This study
ZL556	ZL524, Mu*c*ts Δ*attR*	This study
ZL562	ZL524, Mu*c*ts ΔSGS	This study
ZL573	ZL562, Mu*c*ts *gp23* ::SGS:: *gp24* (SGS at 11.4 kb from L end)	This study
ZL 578	ZL562, Mu*c*ts *gp41*::SGS (SGS at 24 kb from L end)	This study
ZL580	MP1999 ΔMu, *malF*::*loxP*1	This study
ZL 582	ZL580, *malF*::*loxP*2 (2^nd^ site next to *loxP*1)	This study
ZL592	ZL580, *yjcF*::*loxP* (37 kb downstream of *malF*)	This study
ZL594	ZL580, *purH*::*loxP* (37 kb upstream of *malF*)	This study
ZL598	ZL592, *pspG*::SGS	This study
ZL604	ZL524, *himA*::*kan*	This study
ZL614	ZL524, *fis*::*kan*	This study
ZL624	ZL524, *hns*::*kan*	This study
ZL634	ZL524, *hupA*::*kan*	This study
ZL652	ZL524, deletion of 74 bp spacer between *L1* and *L2* sites within Mu *attL*	This study
ZL656	ZL524, Mu*c*ts Δ IHF site	This study
ZL660	ZL524, Mu*c*ts Δ*sis*	This study
ZL662	ZL660, Mu*c*ts Δ*attR*	This study
ZL670	ZL524, Mu*c*ts ΔP*mom*	This study
ZL672	ZL662, Mu*c*ts ΔP*mom*	This study
ZL704	ZL580, *lamB*::*loxP* (5 kb downstream of *malF*)	This study
ZL706	ZL704, *malE*::SGS	This study
ZL708	ZL580, *ubiC*::*loxP* (9 kb downstream of *malF*)	This study
ZL710	ZL708, *lamB*:: SGS	This study
ZL810	ZL580, *dinF*::*loxP*::*yjbJ* (15 kb downstream of *malF*)	This study
ZL712	ZL580, *yjbS*::*loxP*::*aphA* (25 kb downstream of *malF*)	This study
ZL714	ZL712, *plsB*::SGS	This study
ZL720	MP1999, *lamB*::*loxP*, *yjbH*::*loxP*	This study
ZL724	MP1999, *ubiC*::*loxP*, *lysC*::*loxP*::*pgi*	This study
ZL728	MP1999, *yjbS*::*loxP*::*aphA*, *aceA*::*loxP*::*aceK*	This study
ZL732	MP1999, Mu*c*ts *E7*:: *loxP*, *gp49*::*loxP*	This study
ZL808	MG1655, λcI857Cm, *loxP* sites inserted 80 bp and 121 bp outside λ *attL* and *attR*, respectively	This study
ZL901	MG1655, *lacZ*::Mu*c*+ (between 366204–366205 nt)	This study
ZL911	ZL901, *loxP* sites inserted 50 bp and 60 bp outside Mu*c*+ *attL* and *attR*, respectively	This study
ZL921	ZL911, Mu*c*+ ΔSGS	This study
ZL931	ZL911, *hns*::*kan*	This study
ZL936	ZL911, *himA*::*kan*	This study
ZL940	EC1512, *yidX*::*cat-sacB*	This study
ZL941	ZL911, *yidX*::*cat-sacB gyrB402*	This study
ZL951	ZL524, Mu *c*::*kan* ΔPe and ΔPe*	This study
RS005	ZL524, Mu*c*ts Δ10 kb left arm of Mu	This study
RS020	RS005, Mu*c*ts Δ10 kb right arm of Mu	This study
RS025	RS020, Mu*c*ts ΔSGS	This study
RS059	ZL580, *yjbY*::*loxP* (17 kb downstream of *malF*)	This study
RS053	ZL524, Mu*c*ts ΔPe	This study
RS092	ZL524, Mu*c*ts ΔPe*	This study
RS093	ZL524, Mu*c*ts ΔPe ΔPe*	This study
RS033	MP1999, Mu*c*ts *B*::*egfp-B*	This study
RS088	RS033, Mu*c*ts ΔSGS	This study
RS048	RS033, Mu*c*ts ΔPe	This study
RS102	RS033, Mu*c*ts ΔPe*	This study
RS103	RS033, Mu*c*ts ΔPe ΔPe*	This study
RS101	MP1999, Mu*c*ts *E*::*egfp-E*	This study
RS106	RS048, Mu*c*ts SGS::*kan-ccdB*	This study
RS107	RS102, Mu*c*ts SGS::*kan-ccdB*	This study

* Δ, deletion of genes/sites.

:: indicates deletion-substitution when an antibiotic resistance cassette or EGFP is inserted in the indicated gene, but insertion when a site (loxP/SGS) or Mu is inserted. :: placed on both sides of a site indicates insertion of that site between the neighboring genes, the exact location given in Table S1.

**Table 2:** Plasmids.

Plasmid	Expressed protein	Resistance	Replication Origin	Induction	Source (ref.)
pKD4	Source for Kan cassette	Kanamycin	oriR6K gamma		Datsenko & Wanner (2000)
pKD45	Source for Kan/ccdB cassette	Kanamycin	oriR6K gamma	Rhamnose	Kolmsee & Hengge (2011)
pKD46	Lamda Red recombinase	Ampicillin	repA101ts & oriR101	Arabinose	Datsenko & Wanner (2000)
pBAD24		Ampicillin	pBR322 & M13	Arabinose	Guzman et al. (1995)
pBAD24-his-Cre	Cre recombinase	Ampicillin	pBR322 & M13	Arabinose	Ma et al. (2009)
pRHA113	Source for *rhaTRS* locus	Ampicillin	pBR322	Rhamnose	Giacalone et al. (2006)
pRHA113-Cre	Cre recombinase	Ampicillin	pBR322	Rhamnose	This study
pJG8	MuB	Kanamycin	p15A		Ge et al. (2011)
pUC19		Ampicillin			NEB
pEGFP-C1	EGFP	Kanamycin	pBR322		Clontech
pEGFP-MuB	EGFP-MuB	Kanamycin	pBR322		This study
pEGFP-MuE	EGFP-MuE	Kanamycin	pBR322		This study

EGFP fusions at the N-termini of MuB and MuE were constructed by amplifying the corresponding genes from MP1999 and cloning them into BglII - SalI restriction enzyme sites on plasmid pEGFP-C1 (Clontech), which generated a 5-amino acid intervening linker SGLRS. The fused genes were transferred back into the Mu prophage in MP1999 by the λ Red recombination methodology.

A rhamnose-inducible Cre expression vector was constructed by amplifying the gene for this recombinase from pBAD24-his-Cre plasmid and cloning into SalI–XbaI restriction enzyme sites within the *rhaTRS* locus on plasmid pRHA113 [81], to generate plasmid pRHA113-Cre, where Cre expression is driven from the *rhaT* promoter.

### Cre recombination *in vivo*

For experiments using Cre expressed from the pBAD24-Cre (Ara) plasmid [82], M9 glucose minimal media were used in the *in vivo* recombination assay because in LB media, basal level leaky expression of Cre from this plasmid resulted in complete recombination by the time the cultures were grown up after plasmid transformation. For plasmid transformation, overnight (O/N) cell cultures in LB were diluted 1∶100 into 20 ml of the same media, and grown at 30°C for 4–5 hr until OD_600_ reached 0.6. They were washed thrice with ice cold 10% glycerol and brought to the final volume of 200 µl. 40 µl of the cells were electroporated (Biorad Gene pulser, 1.8 kV, 1 mm cuvette) with 90 ng of the plasmid. After recovery for an hour in 1 ml of minimal media at 30°C, a 1∶200 dilution of the culture in the same medium with added ampicillin (100 µg/ml) was propagated at 30°C (Figure S1A). Aliquots at different times of growth were tested for extent of recombination with and without inducer (1 mM arabinose), followed by DNA extraction from 1 ml of culture using the Wizard Genomic DNA purification kit from Promega (Figure S1B). Recombination products were assayed by qPCR as described below. An optimal substrate recombination of ∼25–30% for the wild-type *malF*::Mu substrate (ZL524) was observed either in early- to mid-log phase cultures (OD_600_ 0.5–0.6) with arabinose added for 20 min, or in late-log phase cultures (OD_600_ 1.2–1.3) without added inducer. However, in inducer-added mid-log phase cultures, there were large variations in the recombined fraction in different strains, whereas without added inducer, this fraction was reliably reproducible in late-log cultures of all strains. The latter conditions were therefore chosen for all the experiments reported in this study, except when testing recombination in the gyrase *ts* strain as described below.

Later in the course of this study we acquired a rhamnose-inducible plasmid (Rha), where the basal-level leaky Cre expression was negligible. We confirmed that upon induction of Cre with rhamnose in late-log phase, recombination efficiencies (REs) were comparable to those obtained with uninduced Cre expression from the Ara plasmid (Figure S1C); REs obtained with the rhamnose-induced Cre were comparable between mid- and late-log cultures (Figure S1D).

Cre assays in the gyrase *ts* mutant were carried out with the Rha plasmid. Wild-type Mu*c*+ (ZL911) and its isogenic gyrase *ts* mutant (ZL941) strains transformed with the pRHA113-Cre plasmid were grown in LB media (supplemented with 0.2% glucose) at 26°C until OD_600_ reached 0.5, then either maintained 26°C or shifted to the non-permissive temperature 37°C for 30 min [78], followed by addition of 1 mM rhamnose for 20 min before DNA extraction.

To control for the effect of NAPs on Cre recombination *per se*, RE of *loxP* site pairs placed outside the Mu domain was monitored simultaneously in both wild-type and ΔNAP strains. The REs were not affected by deletion of any of the NAPs tested (Zheng Lou, Ph.D. dissertation).

### Real-time qPCR

Aliquots with 50 ng of DNA, 10 µl SYBR master mix (Applied Biosystems Inc; includes dNTPs, enzyme and buffer), 0.4 µl of each primer (10 µM) and 8.2 µl of double distilled H_2_O were held for 10 min at 95°C, followed by 40 cycles of 15 sec at 95°C and 1 min at 60°C (7900HT; Applied Biosystems). Three independent biological replicates were tested, and for each biological replicate three independent technical replicates were performed. Product integrity was checked using the dissociation curve. Cycle Threshold (C_t_) was read out, and the starting template amount was quantified based on the value of C_t_ assuming exponential growth at early stages of amplification.

Recombination efficiencies were calculated based on the threshold cycle (Ct). The relative threshold cycles of each sample were calculated as, 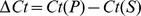 where (P) represents recombination product and (S) the substrate before recombination. The recombination efficiencies (RE) of different samples are normalized to set the recombination efficiency of the control *loxP* sites as 1. The relative recombination efficiency of each sample is calculated as 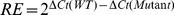. The primer pair for amplifying the starting substrates were (1) ‘RT attL loxP t and RT attL loxP b’ for *malF*::Mu in MP1999, (2) ‘RT LR loxP t and RT LR loxP b’ for *malF* without Mu, (3) ‘RT lacZ L t and RT lacZ L b’ for *lacZ*::Mu, and (4) ‘RT λ L t and RT λ L b’ for λ (other primers are listed in Table S2).

Primer efficiency of a primer pair (say A & B) was determined as follows: Primer A was linked to pUC19t (forward) and primer B was linked to pUC19b (reverse); the pUC primers anneal to pUC19 plasmid and amplify a common 180 bp fragment. The PCR products were purified by Qiaquick PCR purification Kit® (Qiagen) and used as templates for qPCR in the following reaction: 12.5 µl SYBR mix (Qiagen), 0.75 µl each of primer A (10 µM) and primer B (10 µM), 1 µl of template (10 ng/µl) (the 180 bp fragment, as described above) and 10 µl of double distilled H_2_O. PCR cycles were as described above. Another qPCR reaction was performed using the internal primer pair pUC19t and pUC19b. The primer efficiency of primer pairs A/B was calculated as the ratio of Ct values of the PCR product obtained using primer A-pUC19t+primer B-pUC19b to that from primers pUC19t+pUC19b.

### Chromosome Conformation Capture (3C) assay

The methodology was modified from published protocols [83], [84]. An O/N cell culture in Luria broth (LB) was diluted 1∶1000 into 50 ml of fresh medium and grown with shaking at 30°C until OD_600_ reached 0.5–0.6.

#### DNA crosslinking and cell lysis

1.35 ml of formaldehyde (Fisher-Scientific, 37%) was added to the 50 ml cell culture and incubated for 20 min at room temperature with slow shaking. Crosslinking was stopped with addition of 12 ml of 2.5 M glycine for 5 min at room temperature (r.t.). Cells were centrifuged at 5000× g for 10 min at 4°C, washed twice in 10 ml ice-cold PBS pH 7.5, resuspended in 1 ml PBS and divided into 100 µl aliquots in 10 eppendorf tubes. The aliquots were centrifuged at 14000× g 4°C for 5 min and the pellets resuspended in 200 µl of 1× restriction enzyme buffer. The centrifugation step was repeated, and the pellet resuspended in 192 µl digestion buffer (1× restriction enzyme buffer, 1× complete protease inhibitor from Roche). Next, 2 µl of 35 KU/µl Ready-Lyse lysozyme (Epicenter Biotechnologies) was added to the suspension and incubated at r.t. for 20 min. Finally, 6 µl of 10% SDS solution was added, followed by O/N incubation at 37°C.

#### Digestion and ligation

100 µl of the cell lysate prepared above was added to 200 µl digestion buffer (see above) and 30 µl of 20% Triton X-100. After incubation at 37°C for 1.5 hr, restriction digestion was performed thrice at 37°C as follows: 3 µl of 100 U/µl enzyme EcoRI or PstI (New England BioLabs) for 3 hr, another 3 µl of enzyme for 3 hr, and finally another 2 µl of enzyme for O/N incubation.

On the following day, digestion was stopped by addition of 50 µl of 10% SDS, incubated at 65°C for 20 min. Samples were centrifuged at 16,000 g for 5 min at r.t. and their supernatants (∼390 µl) transferred to 15 ml tubes, which contained 4095 µl of pre-ligation buffer (25 µl of 20% Triton X-100, 100 µl of 25× complete protease inhibitor, 50 µl of 1M Tris-HCl pH 7.8, 50 µl of 10 mg/ml BSA, and 3645 µl of H_2_O). After incubation for 1 hr at 37°C with shaking, 500 µl of 10× T4 DNA ligase buffer and 15 µl of 2000 U/µl T4 DNA ligase (New England BioLabs) were added to a final DNA concentration of 0.8 ng/µl. The ligation reaction was incubated at 4°C for 3 days, replenishing ATP each day by addition of 50 µl of 100 mM ATP. Finally, the ligation reaction was incubated for 1 hr at r.t., followed by addition of Proteinase K mixture (210 µl of 5M NaCl, 10 µl of 0.5 M EDTA, 105 µl of 10 mg/ml Proteinase K) to stop the reaction. Crosslinks were reversed by O/N incubation at 65°C.

#### DNA purification

To remove RNA from the samples, 30 µl of 10 mg/ml RNase A (Promega) was added for 45 min at 37°C. DNA was extracted twice by an equal volume of phenol-chloroform-isoamyl alcohol pH 6.7 (Fisher-Scientific), and with pure chloroform once. DNA was precipitated by addition of glycogen (final concentration 50 µg/ml, Affymetrix), 500 µl 3M sodium acetate, and 10 ml isopropyl alcohol, per 5 ml DNA solution. The air-dried DNA pellet was resuspended in 100 µl of 10 mM Tris-HCl pH 7.5 for qPCR quantification as described above. All qPCR results were validated by regular PCR, DNA electrophoresis, and DNA sequencing.

### Cre recombination *in vitro*

Cre recombination was performed on genomic DNA that was crosslinked with formaldehyde and treated as described above, just prior to addition of restriction enzymes. 10 µl of Cre (1 µg/µl) was added for 3 hr at 37°C (∼30% recombination in wild-type prophage DNA substrate). Cre protein was a gift from Dr. Makkuni Jayaram [85]. DNA was treated with RNAse and precipitated as described above under ‘DNA purification’.

### RNA Isolation and RACE

For RNA isolation, cells were grown with shaking at 30°C in 10 ml of LB until OD_600_ reached 0.6. Two ml of culture (∼1×10^8^ cells) were harvested for RNA isolation using ToTALLY RNA Kit from Ambion according to their specification. MICROBExpress Kit from Ambion was used to enrich for mRNA from 10 µg of purified total RNA by removing the 16S and 23S ribosomal RNAs (rRNAs). The final yield of enriched mRNA was ∼1 µg. The quality of total RNA was checked by agarose gel electrophoresis and the RNA concentration was determined by measuring OD at 260 and 280 nm. RNA samples were stored at −80°C until use.

5′-RACE (5′-Rapid Amplification of cDNA Ends) was used to determine the transcriptional start sites, using the SMARTer RACE cDNA Amplification Kit (Clontech, Mountain View, CA). Gene-specific primers (GSP1-3 for Pe* and GSP4-5 for Pe promoter) were used to amplify the 5′-end of isolated mRNA. The RACE PCR amplified bands were gel-purified and sequenced directly.

### Fluorescence measurement of EGFP-MuB/MuE strains

The fluorescence intensity of EGFP strains was recorded using a PTI Quanta Master Model C scanning spectrofluorometer. Strains were sub-cultured by 1∶50 dilution from an O/N culture into 5 ml of LB, and grown at 30°C until OD_600_ reached 0.6. Three ml of the culture cell were placed in a Bio-Rad VersaFluor cuvette with a path length of 10 mm. Excitation and emission wavelengths were set at 488 nm and 507 nm, respectively. Fluoresence measurements were obtained from three independent cultures propagated on different days, each measured in triplicate. AU is arbitrary units. Each fluorescent value was derived by calculating fluorescent data of EGFP-MuB/MuE strain minus fluorescent data of WT strain without EGFP fusion.

### Fluorescence microscopy

2 µl of culture prepared as described above was placed on a glass slide, and examined with Olympus BX53 microscope equipped with a GFP filter. Photographs were taken with Olympus XM10 camera and processed with Photoshop (Adobe Systems, Palo Alto, CA).

## Supporting Information

Figure S1 (A) Growth curves of the wild-type *loxP-malF*::Mu-*loxP* strain ZL524 (MU) at 30°C in M9 glucose minimal media, containing one of two Cre-expressing plasmids. ZL524 contains either the arabinose-inducible Cre plasmid pBAD24-His-Cre (Ara), or the rhamnose-inducible Cre plasmid pRHA113-Cre (Rha). Growth curves are monitored without added inducer. (B) Cre recombination in ZL524 (MU) carrying the Ara plasmid was estimated at different times during the growth curve shown in (A). Recombination was assessed either in the absence (−) or presence (+) of 1 mM arabinose inducer, added for 20 min. Percentage of substrate recombined was estimated by measuring the initial (without Cre plasmid) and final substrate concentration (with Cre plasmid) using qPCR. The data are derived from three technical repeats of three biologically independent samples. (C) Strains containing either the Ara or the Rha plasmid were propagated in minimal media for ∼40 hr. No inducer was added in strains with the Ara plasmid; 1 mM rhamnose was added for 20 min to strains with the Rha plasmid. Recombination efficiency (RE) was calculated as described in Materials and Methods. RE of *loxP* sites flanking the wild-type *malF*::Mu prophage is set to 1 (MU, ZL524), and compared to a pair of *loxP* sites separated by an equivalent 37 kbp on chromosomal DNA in *malF* region (*malF/yjcF*, ZL592). (**D**) As in (**C**), except at ∼30 hr of growth.
(TIF)

Figure S2(A) Position of pairs of *loxP* sites at different distances around the *malF* locus on the *E. coli* chromosome. These *loxP* pairs were engineered after excision of Mu from *malF* in ZL524. Strain numbers for the indicated distances (shown in parentheses) between *loxP* pairs are: *malF*, ZL582 (190 bp); *malF-lamB*, ZL704 (5 kbp); *malF-ubiC*, ZL708 (9 kbp); *malF-yjbJ*, ZL810 (15 kbp); *malF-yjbY*, RS059 (17 kbp); *malF-yjbS*, ZL712 (25 kbp); *malF-yjcF*, ZL592 (37 kbp); *purH-malF*, ZL594 (37 kbp). Strains are listed in Table 1, and the exact position of *loxP* sites is found in Table S1. Primers used are listed in Table S2. (B) The SGS site was engineered at the center of the 37 kbp *yjcF-malF E. coli* DNA segment (see A) and RE of flanking *loxP* sites measured. MU (ZL524), *yjcF/malF* (ZL592), *yjcF/malF*+SGS (ZL598). (C) Effect of SGS on the RE of *loxP* site pairs at varying distances in *E. coli* DNA. The SGS site was introduced at the center of DNA flanked by *loxP* pairs separated by 5–37 kbp shown in A. The RE of these sites is compared in strains with (red) and without (blue) SGS. The strains without SGS are listed in A. Those with added SGS are: ZL706 (5 kbp), ZL710 (9 kbp), ZL714 (25 kbp), ZL598 (37 kbp). (D) Double log plot of RE vs distance as described for the data in Figure 1B, except that the RE value at 37 kbp is omitted. Here, 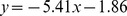.(TIF)

Figure S3 Genetic map of the left end of Mu and identification of a new promoter Pe*. (A) PcM and Pe are divergent promoters that control the lysogeny-lysis decision; PcM drives transcription of the lysogenic repressor gene *c* (Rep protein), and Pe controls a long early transcript from *ner* to *C*, which encodes not only the transposition functions A and B, but also largely uncharacterized functions in the semi-essential (SE) region. Pm and Plys are late promoters active during lytic growth. Pe* is a new promoter identified in this study [49], [86]. (B) EGFP-MuB fluorescence in strains containing Mu prophages without (WT) or with (EGFP-MuB) EGFP fused to the B. Both strains were grown at 30°C, where the prophage does not enter lytic growth. WT (MP1999), EGFP-MuB (RS033). (C) Characterization of the Mu early transcripts in a Mu lysogen. Total RNA was isolated from the uninduced strain MP1999. 5′-RACE-PCR was performed on the first-strand cDNA synthesized from the total RNA using primers within Mu*A* and Mu*B* genes, and the products were directly sequenced to identify the 5′ ends as described in Materials and Methods. Two products were initially obtained – Pe and Pe*. These were characterized separately using gene-specific primers (GSPs) placed are varying distances to confirm that the size of the product varied as predicted from the identity of the 5′ terminus. GSP positions on the Mu genome are shown in the schematic below. Lanes 3 and 7 contain DNA size markers. (D) Position of Pe and Pe* with respect to *ner* and *A* gene ORFs and their homology to the *E. coli* sigma 70 promoter consensus sequence. Transcription start sites as determined by 5′RACE are indicated by magenta coloring of the A nucleotide starts determined for both transcripts. Start of the Pe transcript matches that reported previously by S1 mapping [87]. Conserved nucleotides in both promoters are underlined. Compared to the sigma 70 consensus promoter, Pe* has the same number of conserved nucleotides as found in the Pe i.e. 5/6 at −35 and 4/6 at −10.
(TIF)

Table S1 Exact position of insertions and deletions in Mu and in *E. coli*. * For Mu, the numbers indicate nucleotide positions starting at 1 at the L end of Mu; for *E. coli*, they indicate nucleotide positions starting at 1 on the *E. coli* genome.
(DOCX)

Table S2 Primers used.
(DOCX)
