# Supplementary figures and images for: Transposable Prophage Mu Is Organized as a Stable Chromosomal Domain of E. coli
Source: PLoS Genet. 2013 Nov 7;9(11):e1003902. doi: 10.1371/journal.pgen.1003902 (PMC3820752; doi:10.1371/journal.pgen.1003902)

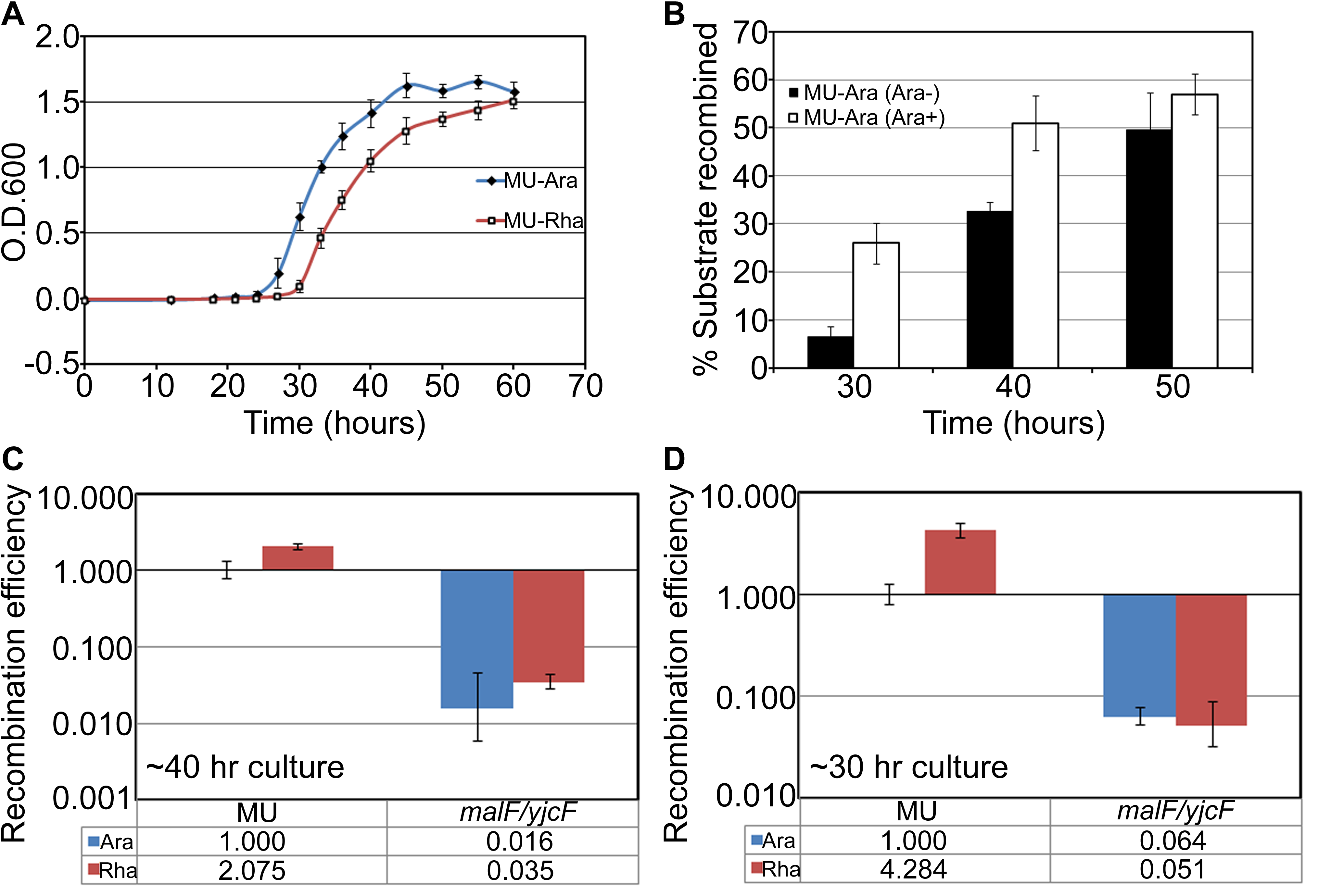

Supplement: Figure S1 — (A) Growth curves of the wild-type loxP-malF::Mu-loxP strain ZL524 (MU) at 30°C in M9 glucose minimal media, containing one of two Cre-expressing plasmids. ZL524 contains either the arabinose-inducible Cre plasmid pBAD24-His-Cre (Ara), or the rhamnose-inducible Cre plasmid pRHA113-Cre (Rha). Growth curves are monitored without added inducer. (B) Cre recombination in ZL524 (MU) carrying the Ara plasmid was estimated at different times during the growth curve shown in (A). Recombination was assessed either in the absence (−) or presence (+) of 1 mM arabinose inducer, added for 20 min. Percentage of substrate recombined was estimated by measuring the initial (without Cre plasmid) and final substrate concentration (with Cre plasmid) using qPCR. The data are derived from three technical repeats of three biologically independent samples. (C) Strains containing either the Ara or the Rha plasmid were propagated in minimal media for ∼40 hr. No inducer was added in strains with the Ara plasmid; 1 mM rhamnose was added for 20 min to strains with the Rha plasmid. Recombination efficiency (RE) was calculated as described in Materials and Methods. RE of loxP sites flanking the wild-type malF::Mu prophage is set to 1 (MU, ZL524), and compared to a pair of loxP sites separated by an equivalent 37 kbp on chromosomal DNA in malF region (malF/yjcF, ZL592). (D) As in (C), except at ∼30 hr of growth. (TIF) [file pgen.1003902.s001.tif]

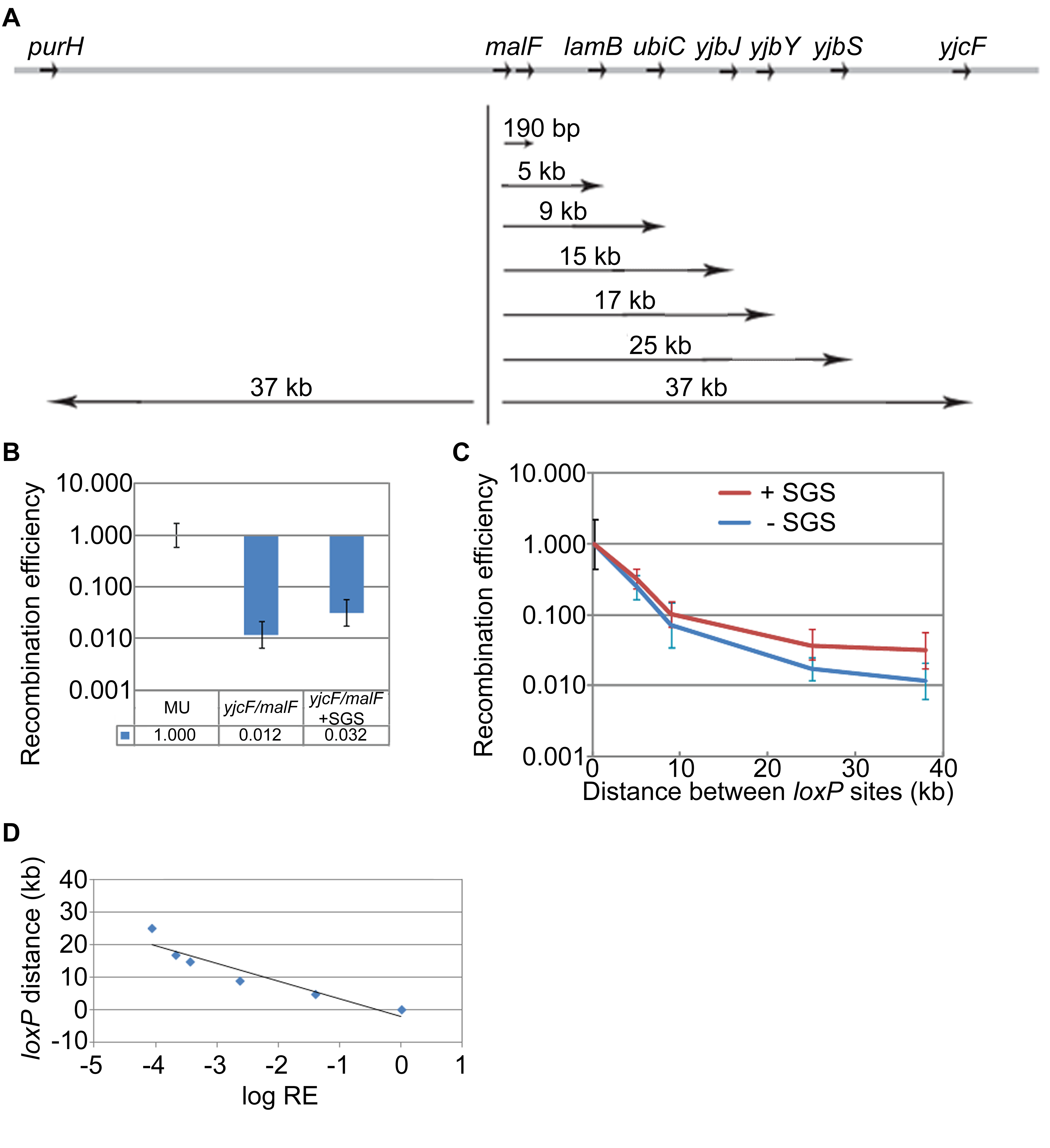

Supplement: Figure S2 — (A) Position of pairs of loxP sites at different distances around the malF locus on the E. coli chromosome. These loxP pairs were engineered after excision of Mu from malF in ZL524. Strain numbers for the indicated distances (shown in parentheses) between loxP pairs are: malF, ZL582 (190 bp); malF-lamB, ZL704 (5 kbp); malF-ubiC, ZL708 (9 kbp); malF-yjbJ, ZL810 (15 kbp); malF-yjbY, RS059 (17 kbp); malF-yjbS, ZL712 (25 kbp); malF-yjcF, ZL592 (37 kbp); purH-malF, ZL594 (37 kbp). Strains are listed in Table 1, and the exact position of loxP sites is found in Table S1. Primers used are listed in Table S2. (B) The SGS site was engineered at the center of the 37 kbp yjcF-malF E. coli DNA segment (see A) and RE of flanking loxP sites measured. MU (ZL524), yjcF/malF (ZL592), yjcF/malF+SGS (ZL598). (C) Effect of SGS on the RE of loxP site pairs at varying distances in E. coli DNA. The SGS site was introduced at the center of DNA flanked by loxP pairs separated by 5–37 kbp shown in A. The RE of these sites is compared in strains with (red) and without (blue) SGS. The strains without SGS are listed in A. Those with added SGS are: ZL706 (5 kbp), ZL710 (9 kbp), ZL714 (25 kbp), ZL598 (37 kbp). (D) Double log plot of RE vs distance as described for the data in Figure 1B, except that the RE value at 37 kbp is omitted. Here, . (TIF) [file pgen.1003902.s002.tif]

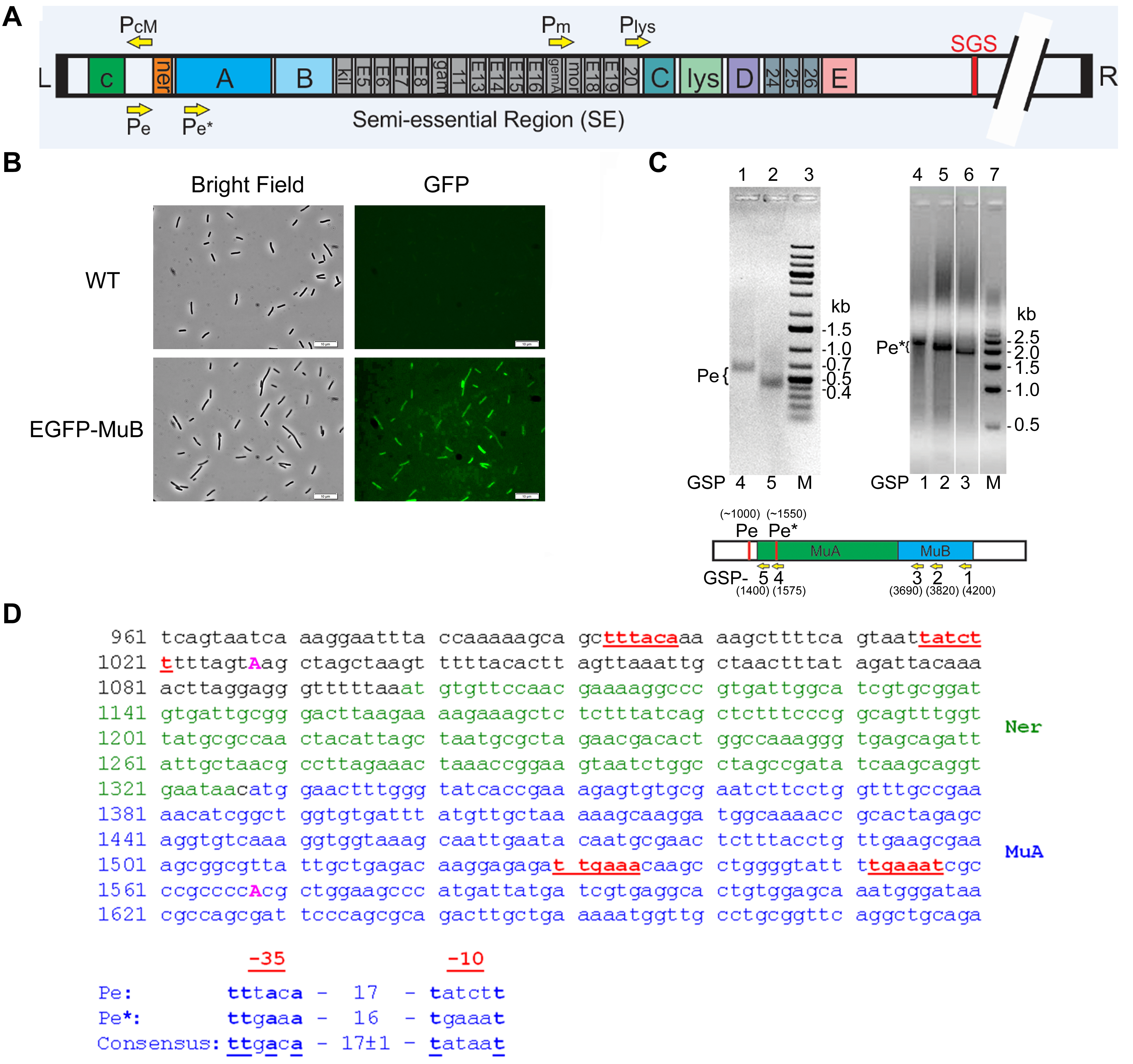

Supplement: Figure S3 — Genetic map of the left end of Mu and identification of a new promoter Pe*. (A) PcM and Pe are divergent promoters that control the lysogeny-lysis decision; PcM drives transcription of the lysogenic repressor gene c (Rep protein), and Pe controls a long early transcript from ner to C, which encodes not only the transposition functions A and B, but also largely uncharacterized functions in the semi-essential (SE) region. Pm and Plys are late promoters active during lytic growth. Pe* is a new promoter identified in this study [49], [86]. (B) EGFP-MuB fluorescence in strains containing Mu prophages without (WT) or with (EGFP-MuB) EGFP fused to the B. Both strains were grown at 30°C, where the prophage does not enter lytic growth. WT (MP1999), EGFP-MuB (RS033). (C) Characterization of the Mu early transcripts in a Mu lysogen. Total RNA was isolated from the uninduced strain MP1999. 5′-RACE-PCR was performed on the first-strand cDNA synthesized from the total RNA using primers within MuA and MuB genes, and the products were directly sequenced to identify the 5′ ends as described in Materials and Methods. Two products were initially obtained – Pe and Pe*. These were characterized separately using gene-specific primers (GSPs) placed are varying distances to confirm that the size of the product varied as predicted from the identity of the 5′ terminus. GSP positions on the Mu genome are shown in the schematic below. Lanes 3 and 7 contain DNA size markers. (D) Position of Pe and Pe* with respect to ner and A gene ORFs and their homology to the E. coli sigma 70 promoter consensus sequence. Transcription start sites as determined by 5′RACE are indicated by magenta coloring of the A nucleotide starts determined for both transcripts. Start of the Pe transcript matches that reported previously by S1 mapping [87]. Conserved nucleotides in both promoters are underlined. Compared to the sigma 70 consensus promoter, Pe* has the same number of conserved nucleotides as found in the Pe i [file pgen.1003902.s003.tif]
