# Supplementary material for: Transposable Prophage Mu Is Organized as a Stable Chromosomal Domain of E. coli
Source: PLoS Genet. 2013 Nov 7;9(11):e1003902. doi: 10.1371/journal.pgen.1003902 (PMC3820752; doi:10.1371/journal.pgen.1003902)
Supplement: Table S1 — Exact position of insertions and deletions in Mu and in E. coli. * For Mu, the numbers indicate nucleotide positions starting at 1 at the L end of Mu; for E. coli, they indicate nucleotide positions starting at 1 on the E. coli genome. (DOCX) [file pgen.1003902.s004.docx]

**Table S1. Exact position of insertions and deletions in Mu and *E. coli***

| **Mu mutations*** | **Position** |
| --- | --- |
| SGS site | 17774-18000 (for addition or deletion) |
| *∆sis* | 35155-35215 |
| ∆*attL* | 1-180 |
| ∆*attR* | 36538-36717 |
| ∆IHF site | 947-958 |
| ∆HU site | 31-104 |
| ∆P*mom* | 35564-35779 |
| ∆Pe | 991-1030 |
| ∆Pe* | 1526-1561 |
| ∆10 kb left arm of Mu | 4315 to 14554 |
| ∆10 kb right arm of Mu | 21429 to 31458 |
| *gp23*::SGS::*gp24* | 11414-11474 replaced by SGS |
| *gp41*::SGS | 23987-24043 replaced by SGS |
| *E7*:: *loxP* | 5111-5171 replaced by *loxP* |
| *gp49*::*loxP* | 31507-31571 replaced by *loxP* |
|  |  |
| ***E. coli* mutations*** | **Position** |
| *lacZ*::Mu*c*^+^ | Mu insertion between 365204-365205^1^ |
| *malF*::*loxP* 1 | 4242073-4242233 replaced by *loxP* |
| *malF*::*loxP* 2 | 4241803-4241883 replaced by *loxP* |
| *lamB*::*loxP* | 4246725-4246783 replaced by *loxP* |
| *ubiC*::*loxP* | 4250927-4250976 replaced by *loxP* |
| *dinF*::*loxP*::*yjbJ* | 4257145-4257194 replaced by *loxP* |
| *yjbS*::*loxP*::*aphA* | 4267036-4267089 replaced by *loxP* |
| *yjcF*::*loxP* | 4279831-4280154 replaced by *loxP* |
| *malE*::SGS | SGS inserted at 4244289 |
| *lamB*::SGS | 4246725-4246783 replaced by SGS |
| *plsB*::SGS | 4253635-4253674 replaced by SGS |
| *pspG*::SGS | 4260863-4261094 replaced by SGS |
| *yjbH*::*loxP* | 4237001-4237170 replaced by *loxP* |
| *lysC*::*loxP*::*pgi* | 4231471-4231650 replaced by *loxP* |
| *aceA*::*loxP*::*aceK* | 4216486-4216570 replaced by *loxP* |
| *purH:loxP* | 4204046-4204952 replaced by *loxP* |
|  | |

^1^ *lacZ*::Mu*c*^+^ strain has another Mu insertion at an unknown location
