# Supplementary material for: Transposable Prophage Mu Is Organized as a Stable Chromosomal Domain of E. coli
Source: PLoS Genet. 2013 Nov 7;9(11):e1003902. doi: 10.1371/journal.pgen.1003902 (PMC3820752; doi:10.1371/journal.pgen.1003902)
Supplement: Table S2 — Primers used. (DOCX) [file pgen.1003902.s005.docx]

**Table S2. Primers used.**

| **Primers for making mutations** | |
| --- | --- |
| **Name** | **Sequence** |
| attL loxP 1t | cgcggtgccatcgttcatttcaatcttgattttcaaagggttgtttaacctgtgacggaagatcacttcg |
| attL loxP 1b | tttaatcagcagcggcagcgtaatcttaaagaagttctggaacggacctgatcaaagggaaaactgtccatat |
| attL loxP 2t | cgcggtgccatcgttcatttcaatcttgattttcaaagggttgtttaaccataacttcgtataatgtatgctatacgaagttat |
| attL loxP 2b | tttaatcagcagcggcagcgtaatcttaaagaagttctggaacggacctgataacttcgtatagcatacattatacgaagttat |
| attR loxP 1t | tttaactttaacaacttcgtgctgattcaactgttaaccaacggcggccctgtgacggaagatcacttcg |
| attR loxP 1b | cgccttcaaaagcgatgcggtaggtgtagttaacaagcaggtcggtataaatcaaagggaaaactgtccatat |
| attR loxP 2t | tttaactttaacaacttcgtgctgattcaactgttaaccaacggcggcccataacttcgtataatgtatgctatacgaagttat |
| attR loxP 2b | cgccttcaaaagcgatgcggtaggtgtagttaacaagcaggtcggtataaataacttcgtatagcatacattatacgaagttat |
| malF loxP 1t | ttttaccagtccattaccgccgacggcaactggggtgatgaaaagctaagtgtgacggaagatcacttcg |
| malF loxP 1b | gttaaaaagacagtgatcagcgagaacaccacggtccagacgaaaatggcatcaaagggaaaactgtccatat |
| malF loxP 2t | ttttaccagtccattaccgccgacggcaactggggtgatgaaaagctaagataacttcgtataatgtatgctatacgaagttat |
| malF loxP 2b | gttaaaaagacagtgatcagcgagaacaccacggtccagacgaaaatggcataacttcgtatagcatacattatacgaagttat |
| 5kR loxP 1t | atactcagagtgtcctgaagggctttaacaagtttgttgttcagtacgcttgtgacggaagatcacttcg |
| 5kR loxP 1b | aaatgcaacgccagaaccctgcgacagccctttaccctgcgaggtcatcgatcaaagggaaaactgtccatat |
| 5kR loxP 2t | atactcagagtgtcctgaagggctttaacaagtttgttgttcagtacgctataacttcgtataatgtatgctatacgaagttat |
| 5kR loxP 2b | aaatgcaacgccagaaccctgcgacagccctttaccctgcgaggtcatcgataacttcgtatagcatacattatacgaagttat |
| 9kR loxP 1t | tcacatcatcgacattaacccgggactttattgagataggccgtgatgcc tgtgacggaagatcacttcg |
| 9kR loxP 1b | ttagtacaacggtgacgccggtaaaaacagttctgttagcaacagcggtt atcaaagggaaaactgtccatat |
| 9kR loxP 2t | tcacatcatcgacattaacccgggactttattgagataggccgtgatgcc ataacttcgtataatgtatgctatacgaagttat |
| 9kR loxP 2b | ttagtacaacggtgacgccggtaaaaacagttctgttagcaacagcggtt ataacttcgtatagcatacattatacgaagttat |
| 15kR loxP 1t | ctatctggcggcgtcactggcgcaatggtacctggtttgccgcaacgtga tgtgacggaagatcacttcg |
| 15kR loxP 1b | gttaggttgtgaatgagagtacgttcacttttcttctgaacgtgagatta atcaaagggaaaactgtccatat |
| 15kR loxP 2t | ctatctggcggcgtcactggcgcaatggtacctggtttgccgcaacgtga ataacttcgtataatgtatgctatacgaagttat |
| 15kR loxP 2b | gttaggttgtgaatgagagtacgttcacttttcttctgaacgtgagatta ataacttcgtatagcatacattatacgaagttat |
| 25kR loxP 1t | tcaacaggtaaagatgtcgttttgttgctattcacatatgatatattcat tgtgacggaagatcacttcg |
| 25kR loxP 1b | acagcattacaaaaacaatcgaagtttataaagatgatttctgattgacc atcaaagggaaaactgtccatat |
| 25kR loxP 2t | tcaacaggtaaagatgtcgttttgttgctattcacatatgatatattcat ataacttcgtataatgtatgctatacgaagttat |
| 25kR loxP 2b | acagcattacaaaaacaatcgaagtttataaagatgatttctgattgacc ataacttcgtatagcatacattatacgaagttat |
| 37kR loxP 1t | cgcggttaacaaatacgttcctcgaaatggttatatgtaccgactctaattgtgacggaagatcacttcg |
| 37kR loxP 1b | cagtaatcttcatagcgactgcatgaagattatcctcagtttgtgacatatcaaagggaaaactgtccatat |
| 37kR loxP 2t | cgcggttaacaaatacgttcctcgaaatggttatatgtaccgactctaatataacttcgtataatgtatgctatacgaagttat |
| 37kR loxP 2b | cagtaatcttcatagcgactgcatgaagattatcctcagtttgtgacatataacttcgtatagcatacattatacgaagttat |
| 2.5kR SGS 1t | ccagtttatccggatgctcaacggtgactttaattccggtatctttctcgtgtgacggaagatcacttcg |
| 2.5kR SGS 1b | attaacggcgataaaggctataacggtctcgctgaagtcggtaagaaattatcaaagggaaaactgtccatat |
| 2.5kR SGS 2t | ccagtttatccggatgctcaacggtgactttaattccggtatctttctcggcccggcaggcgttcctgcg |
| 2.5kR SGS 2b | attaacggcgataaaggctataacggtctcgctgaagtcggtaagaaattttgcgggaggtgcgggaaaa |
| 5kR SGS 2t | atactcagagtgtcctgaagggctttaacaagtttgttgttcagtacgctgcccggcaggcgttcctgcg |
| 5kR SGS 2b | cgcagcatgtgaccgttgttgttgatattgtaggcaaatttttcgttatcttgcgggaggtgcgggaaaa |
| 12kR SGS 1t | gcgagcagcttattaaacagatcctgacgagcaggcaaacgtgggcctactgtgacggaagatcacttcg |
| 12kR SGS 1b | atggcggatgaacacggcacggataaaactatcgctcagaaactggcgcgatcaaagggaaaactgtccatat |
| 12kR SGS 2t | gcgagcagcttattaaacagatcctgacgagcaggcaaacgtgggcctacgcccggcaggcgttcctgcg |
| 12kR SGS 2b | atggcggatgaacacggcacggataaaactatcgctcagaaactggcgcgttgcgggaggtgcgggaaaa |
| 18kR SGS 1t | ggcatgattcttgtaatgccagcaagagatttcatatttgggagagcatctgtgacggaagatcacttcg |
| 18kR SGS 1b | ccctttctttttatagttcggctgtatgtagggtacagcacgatgaatctatcaaagggaaaactgtccatat |
| 18kR SGS 2t | ggcatgattcttgtaatgccagcaagagatttcatatttgggagagcatcgcccggcaggcgttcctgcg |
| 18kR SGS 2b | ccctttctttttatagttcggctgtatgtagggtacagcacgatgaatctttgcgggaggtgcgggaaaa |
| 5kL loxP 1t | atgggaacggcagatttgtggctgacggaccatctgctgaccaccggcag tgtgacggaagatcacttcg |
| 5kL loxP 1b | acatagacatcgttctgcacatactcgcgcacatgggtacgcacgcgcgg atcaaagggaaaactgtccatat |
| 5kL loxP 2t | atgggaacggcagatttgtggctgacggaccatctgctgaccaccggcag ataacttcgtataatgtatgctatacgaagttat |
| 5kL loxP 2b | acatagacatcgttctgcacatactcgcgcacatgggtacgcacgcgcgg ataacttcgtatagcatacattatacgaagttat |
| 10kL loxP 1t | gcaccccctcaggtgttatcacaggactggctcctccaacaccgttactt tgtgacggaagatcacttcg |
| 10kL loxP 1b | tagctgaactactgaccgccaggagtggatgaaaaatccgcatgacccca atcaaagggaaaactgtccatat |
| 10kL loxP 2t | gcaccccctcaggtgttatcacaggactggctcctccaacaccgttactt ataacttcgtataatgtatgctatacgaagttat |
| 10kL loxP 2b | tagctgaactactgaccgccaggagtggatgaaaaatccgcatgacccca ataacttcgtatagcatacattatacgaagttat |
| 25kL loxP 1t | gcaacaacaaccgttgctgactgtaggccggataaggcgttcacgccgca tgtgacggaagatcacttcg |
| 25kL loxP 1b | cttatccagcctacgttcggcaacggctgtaggcatgataagacgcgcaa atcaaagggaaaactgtccatat |
| 25kL loxP 2t | gcaacaacaaccgttgctgactgtaggccggataaggcgttcacgccgca ataacttcgtataatgtatgctatacgaagttat |
| 25kL loxP 2b | cttatccagcctacgttcggcaacggctgtaggcatgataagacgcgcaa ataacttcgtatagcatacattatacgaagttat |
| 37kL loxP 1t | ttagcagaaaaaggtctgccggtaaccgaagtttccgattacaccggttttgtgacggaagatcacttcg |
| 37kL loxP 1b | cgcaataatcacttcgacaaactgacgagaaatgatggcctgtgcggtttatcaaagggaaaactgtccatat |
| 37kL loxP 2t | ttagcagaaaaaggtctgccggtaaccgaagtttccgattacaccggtttataacttcgtataatgtatgctatacgaagttat |
| 37kL loxP 2b | cgcaataatcacttcgacaaactgacgagaaatgatggcctgtgcggtttataacttcgtatagcatacattatacgaagttat |
| del SGS 1t | gtggggcggtacacccgatatggcaccccgcccggcaggcgttcctgcgc tgtgacggaagatcacttcg |
| del SGS 1b | gctgctcatcatggtttgtgagcagtcagtttgcgggaggtgcgggaaaa atcaaagggaaaactgtccatat |
| del SGS 2t | acacccgatatggcaccccgcccggcaggcgttcctgcgcttttcccgca |
| del SGS 2b | tggtttgtgagcagtcagtttgcgggaggtgcgggaaaagcgcaggaacg |
| del sis 1t | atgtaagggtatcaacaaatgaccagaatacagacctgcaacgaaacgcttgtgacggaagatcacttcg |
| del sis 1b | cagtgtgtcacctttttgaaggcgctttaaagcgcgttttaatcccggtcatcaaagggaaaactgtccatat |
| del sis 2t | atcaacaaatgaccagaatacagacctgcaacgaaacgctgaccgggatt |
| del sis 2b | cctttttgaaggcgctttaaagcgcgttttaatcccggtcagcgtttcgtt |
| del pmom 1t | ggaatcccccgcaagcaggttgcattgatctacgatgtggccctgtcaac tgtgacggaagatcacttcg |
| del pmom 1b | cgcggacacctgatttcaatgtgatcaaaggaatccgccttaaataacag atcaaagggaaaactgtccatat |
| del pmom 2t | gcaagcaggttgcattgatctacgatgtggccctgtcaacctgttattta |
| del pmom 2b | caaaggaatccgccttaaataacaggttgacagggccacatcgtagatca |
| del IHF site 1t | agtattattcttttctataaagttacttttcaaaatttaaactccttatttgtgacggaagatcacttcg |
| del IHF site 1b | aaaagctttttgtaaagctgctttttggtaaattcctttgattactgattatcaaagggaaaactgtccatat |
| del IHF site 2t | agtattattcttttctataaagttacttttcaaaatttaaactccttattaatcagtaatcaaaggaatttaccaaaaag |
| del IHF site 2b | aaaagctttttgtaaagctgctttttggtaaattcctttgattactgattaataaggagtttaaattttgaaaagtaact |
| del attR 1t | caaggttcagccataccctaagtgatccccatgtaatgaataaaaagcag tgtgacggaagatcacttcg |
| del attR 1b | gggccgccgttggttaacagttgaatcagcacgaagttgttaaagttaaa atcaaagggaaaactgtccatat |
| del attR 2t | caaggttcagccataccctaagtgatccccatgtaatgaataaaaagcagtttaactttaacaacttcgt |
| del attR 2b | gggccgccgttggttaacagttgaatcagcacgaagttgttaaagttaaactgctttttattcattacat |
| del attL 1t | caggtccgttccagaacttctttaagattacgctgccgctgctgattaaa tgtgacggaagatcacttcg |
| del attL 1b | gttatcggttttgaacgttttttgaagctgttattgaaatgatttgcagtatcaaagggaaaactgtccatat |
| del attL 2t | caggtccgttccagaacttctttaagattacgctgccgctgctgattaaaactgcaaatcatttcaataa |
| del attL 2b | gttatcggttttgaacgttttttgaagctgttattgaaatgatttgcagttttaatcagcagcggcagcg |
| del HU site 1t | tgattaaaccgctgacgccg tgtattgattcacttgaagtacgaaaaaaatgtgacggaagatcacttcg |
| del HU site 1b | aacaaaagcaattttttactatctttcgcgtttcattgattaacgactaaatcaaagggaaaactgtccatat |
| del HU site 2t | tgtattgattcacttgaagtacgaaaaaaattagtcgttaatcaatgaaa |
| del HU site 2b | aacaaaagcaattttttactatctttcgcgtttcattgattaacgactaa |
| del MuA t | aaactaaaccggaagtaatctggcctagccgatatcaagcaggtgaataa gtgtaggctggagctgcttc |
| del MuA b | ggaaatattcatctgaataacctcgtaattaaattttgtttaaacgttaa catatgaatatcctcctta |
| del himA t | atggcgcttacaaaagctgaaatgtcagaatatctgtttgataagcttgtgtaggctggagctgcttc |
| del himA b | ttactcgtctttgggcgaagcgttttcgacccggctttttaacttctgcatatgaatatcctccta |
| del MuB t | aaagccatttaattaacgtttaaacaaaatttaattacgaggttattcag gtgtaggctggagctgcttc |
| del MuB b | gcatccgttgccatttttatatttcgggccatcataatttcttctccttc catatgaatatcctcctta |
| del fis t | ggcatacttcgaaaattttgcgtaaacagaaataaagagctgacagaact gtgtaggctggagctgcttc |
| del fis b | ttccccatgccgagtagcgcctttttaatcaagcatttagctaacctgaacatatgaatatcctcctta |
| del hupA t | ccgtcgcactcgatgcttagcaagcgataaacacattgtaaggataactt gtgtaggctggagctgcttc |
| del hupA b | aaaaggggtgaaaccaccccttcgttaaaactgttcactgccacgcaatc catatgaatatcctcctta |
| del hns t | tctattattacctcaacaaaccaccccaatataagtttgagattactaca gtgtaggctggagctgcttc |
| del hns b | aaaaaatcccgccgctggcgggattttaagcaagtgcaatctacaaaaga catatgaatatcctcctta |
| SGS L 1t | tacagaactgacggaagcaacgcccgttccgcccatgccacagcccctgatgtgacggaagatcacttcg |
| SGS L 1b | aaatttctttccttcggatctcgtgttcccggactgatgccttgtcgcggatcaaagggaaaactgtccatat |
| SGS L 2t | tacagaactgacggaagcaacgcccgttccgcccatgccacagcccctgaaggcataaaatcagccgcacagattttttaaaacgcgccacggga |
| SGS L 2b | aaatttctttccttcggatctcgtgttcccggactgatgccttgtcgcggacaccgttaaataccggtttaaaaatcccgtggcgcgttttaaaa |
| SGS R 1t | cggcatcgctgacacaagaacgggggcgagtggctgcggtgccggaatga tgtgacggaagatcacttcg |
| SGS R 1b | ttttagcctgctggcagtgccggaaaaagcgggacagcgggagagacaag atcaaagggaaaactgtccatat |
| SGS R 2t | cggcatcgctgacacaagaacgggggcgagtggctgcggtgccggaatga aggcataaaatcagccgcacagattttttaaaacg cgccacggga |
| SGS R 2b | ttttagcctgctggcagtgccggaaaaagcgggacagcgggagagacaag acaccgttaaataccggtttaaaaatcccgtggcgcgttttaaaa |
| Mu L loxP 1t | aatgtcaaaatccgaaatgaaattcaggcattaatccgaattcaggatgtgacggaagatcacttcg |
| Mu L loxP 1b | cgcacagatgctgtaatggtcgaaagtaatcaggtaagttttttcaccatatcaaagggaaaactgtccatat |
| Mu L loxP 2t | aatgtcaaaatccgaaatgaaattcaggcattaatccgaattcaggaataacttcgtataatgtatgctatacgaagttat |
| Mu L loxP 2b | cgcacagatgctgtaatggtcgaaagtaatcaggtaagttttttcaccatataacttcgtatagcatacattatacgaagttat |
| Mu R loxP 1t | atgttttatattgataacgacagcggcgtaaccgtcatgccgcccgtatctgtgacggaagatcacttcg |
| Mu R loxP 1b | gcgtgtttaataactccgcctgcacaatattaaaccagtccatgccgggcatcaaagggaaaactgtccatat |
| Mu R loxP 2t | atgttttatattgataacgacagcggcgtaaccgtcatgccgcccgtatcataacttcgtataatgtatgctatacgaagttat |
| Mu R loxP 2b | gcgtg tttaa taact ccgcc tgcacaatattaaaccagtccatgccgggc ataacttcgtatagcatacattatacgaagttat |
| λ L loxP 1t | gggatgcaaaatagtgttgagcatcgaaattctgcgcttcttttgccgactgtgacggaagatcacttcg |
| λ L loxP 1b | tagcccggagcaacctgtgaacacattttcagtttcccgtctggcgctggatcaaagggaaaactgtccatat |
| λ L loxP 2t | gggatgcaaaatagtgttgagcatcgaaattctgcgcttcttttgccgacataacttcgtataatgtatgctatacgaagttat |
| λ L loxP 2b | tagcccggagcaacctgtgaacacattttcagtttcccgtctggcgctggataacttcgtatagcatacattatacgaagttat |
| λ R loxP 1t | catctggcagagtgattaactaaacatcgcagtaatcgaggcgcttgccatgtgacggaagatcacttcg |
| λ R loxP 1t | tgatacccgcgtattaccgcaagggtttggctctggtctggtagcaatgcatcaaagggaaaactgtccatat |
| λ R loxP 1t | catctggcagagtgattaactaaacatcgcagtaatcgaggcgcttgccaataacttcgtataatgtatgctatacgaagttat |
| λ R loxP 1t | tgatacccgcgtattaccgcaagggtttggctctggtctggtagcaatgcataacttcgtatagcatacattatacgaagttat |
| lacZ L loxP 1t | cagcgcagcaccatcaccgcgaggcggttttctccggcgcgtaaaaatgctgtgacggaagatcacttcg |
| lacZ L loxP 1b | atccgacgggttgttactcgctcacatttaatgttgatgaaagctggctaatcaaagggaaaactgtccatat |
| lacZ L loxP 2t | cagcgcagcaccatcaccgcgaggcggttttctccggcgcgtaaaaatgcataacttcgtataatgtatgctatacgaagttat |
| lacZ L loxP 2b | atccgacgggttgttactcgctcacatttaatgttgatgaaagctggctaataacttcgtatagcatacattatacgaagttat |
| lacZ R loxP 1t | gcgcatcgtaaccgtgcatctgccagtttgaggggacgacgacagtatcg tgtgacggaagatcacttcg |
| lacZ R loxP 1b | gactgggaaaaccctggcgttacccaacttaatcgccttgcagcacatcc atcaaagggaaaactgtccatat |
| lacZ R loxP 2t | gcgcatcgtaaccgtgcatctgccagtttgaggggacgacgacagtatcgataacttcgtataatgtatgctatacgaagttat |
| lacZ R loxP 2b | gactgggaaaaccctggcgttacccaacttaatcgccttgcagcacatccataacttcgtatagcatacattatacgaagttat |
| yidx catsac t | gtagtaccagcgtgatgacgttcgcgtttgccgtgcgtgtaatgtagtactgtgacggaagatcacttcg |
| yidx catsac b | cagatttctgcccgacgcatgtcatttttttatgcattgttctttttgtgatcaaagggaaaactgtccatat |
| 10kb L-FR | tgaattagacctggacgttgatatttcaacgctgctgcgtaattaagaaggagaagaaattcagaagaactcgtcaagaa |
| 10kb L-RV | ggctctgcatttcatcgtcaaaatcaaacggctgcccggaaatatccagaatacggcccacgtcatcgccattaattcac |
| 10kb L-fill-FR | tgaattagacctggacgttgatatttcaacgctgctgcgtaattaagaaggagaagaaattcagggcgtcttatatgaat |
| 10kb L-fill-RV | catgaccattgccagctcat |
| 10kb R-FR | tcccggacgaaaccccggaatttgccgcacatatcaatgtgaatcaggaaaaggatcatgtcagaagaactcgtcaagaa |
| 10kb R-RV | tacgctgggcggatacgggcggcatgacggttacgccgctgtcgttatcaatataaaacacgtcatcgccattaattcac |
| 10kb R-fill-FR | tcccggacgaaaccccggaatttgccgcacatatcaatgtgaatcaggaaaaggatcatggcgctggaatgtttactgga |
| 10kb R-fill-RV | gaaaaccagcgaacgatagc |
| 17kb 2LoxP-FR | tcgatagtatggaaggcattatataaaaggacccaatatttattgggtataacttcgtataatgtatgctatacgaagttattcagaagaactcgtcaag |
| 17kb 2LoxP-RV | ttcaaatgtagagattattttatcgatatatcatggggttatgttattcacgtcatcgccattaattcac |
| dSGS FR | gggaacaggaaatttttgatgccatcagaaaacgcgtcagcgccgctctgaggcaataaatcagaagaactcgtcaagaa |
| dSGS RV | aaacgggattcatacaccgttaaataccggtttaaaaatcccgtggcgcgttttaaaaaacgtcatcgccattaattcac |
| dSGS fill-FR | gggaacaggaaatttttgatgccatcagaaaacgcgtcagcgccgctctgaggcaataaacagaatcagattttttaaaacgcgcc |
| dSGS fill-RV | ggagaaagaaagtgaaaggaag |
| dPe FR | atttaaactccttatttatcaacgcgttaatcagtaatcaaaggaatttaccaaaaagcatcagaagaactcgtcaagaa |
| dPe RV | cctcctaagttttgtaatctataaagttagcaatttaactaagtgtaaaaacttagctagcgtcatcgccattaattcac |
| dPe fill-FR | atttaaactccttatttatcaacgcgttaatcagtaatcaaaggaatttaccaaaaagcactagctaagtttttacactt |
| dPe fill-RV | gttggaacacatttaaaaac |
| dMuB FR | cagacgtaaaaaagccatttaattaacgtttaaacaaaatttaattacgaggttattcagtcagaagaactcgtcaagaa |
| dMuB RV | ccagttctgcgcatccgttgccatttttatatttcgggccatcataatttcttctccttccgtcatcgccattaattcac |
| EGFP-MuB FR | aaagccatttaattaacgtttaaacaaaatttaattacgaggttattcagatggtgagcaagggcgagga |
| EGFP-MuB RV | gcatccgttgccatttttatatttcgggccatcataatttcttctccttcttaattacgcagcagcgttg |
| MuB-EcoRI-FR | gctcaagcttcgaattctatgaatatttccgatattcgcg |
| MuB-SacII-FR | gacggtaccgcgggcatgaatatttccgatattcgcg |
| MuB-BamHI-RV | atcgttggatccttaattacgcagcagcgttg |
| dPe* FR | cgaactctttacctgttgaagcgaaagcggcgttattgctgagacaaggatcagaagaactcgtcaagaa |
| dPe* RV | ttgctccacagtgcctcacgatcataatcatgggcttccagcgtggggcgcgtcatcgccattaattcac |
| dPe* fill-FR | cgaactctttacctgttgaagcgaaagcggcgttattgctgagacaaggacgccccacgctggaagc |
| dPe* fill-RV | ttatcccatttgctccacag |
| dMuE FR | cgcgttgccggtgtcaagcgtcctcgtccccgtgcatgacaggaggcgtgtcagaagaactcgtcaagaa |
| dMuE RV | ggggcgcatgaagggctttaaggttgttttctctggtgttcataaccctccgtcatcgccattaattcac |
| EGFP-MuE FR | cgcgttgccggtgtcaagcgtcctcgtccccgtgcatgacaggaggcgtgatggtgagcaagggcgag |
| EGFP-MuE RV | ggggcgcatgaagggctttaaggttgttttctctggtgttcataaccctctcatgccttacccagcag |
| MuE BglII-FR | ggactcagatctatggacaggaaaacacgg |
| MuE SalI-RV | tctgcagtcgactcatgccttacccagcag |
| **Primers for real-time PCR** | |
| **Name** | **Sequence** |
| RT attL loxP t | ctacgcggtgccatcgtt |
| RT attL loxP b | acggcgtcagcggtttaa |
| RT LR loxP t | tcgcgtcctgctgattctg |
| RT LR loxP b | aaagcgatgcggtaggtgtag |
| RT malF b | gttaaaaagacagtgatcag |
| RT 5kR b | aaatgcaacgccagaaccct |
| RT 9KR b | ttagtacaacggtgacgccg |
| RT 15KR b | gttaggttgtgaatgagagt |
| RT 25KR b | tacagcagataaatgttcta |
| RT 37KR b | agcgactgcatgaagattat |
| RT 37kL t | aaggtctgccggtaaccgaa |
| RT 5kL t | atgggaacggcagatttgtg |
| RT 5kL b | acatagacatcgttctgcac |
| RT 10kL t | gcaccccctcaggtgttatc |
| RT 10kL b | tagctgaactactgaccgcc |
| RT 25kL t | gcaacaacaaccgttgctga |
| RT 25kL b | cttatccagcctacgttcgg |
| RT lacZ L t | cagcgcagcaccatcaccgc |
| RT lacZ L b | atccgacgggttgttactcg |
| RT lacZ R b | gactgggaaaaccctggcg |
| RT λ L t | gggatgcaaaatagtgttga |
| RT λ L b | tagcccggagcaacctgtga |
| RT λ R b | tgatacccgcgtattaccgc |
| 17 L Top FR | tgctgattctgccctacg |
| 17 L Bot RV | cgccttcaaaagcgatgc |
| 17 LR Bot RV | tcccgattcgcagcgcat |

| **Primers for primer efficiency test** | |
| --- | --- |
| **Name** | **Sequence** |
| pUC19 t | tgcgcaacgttgttgccatt |
| pUC19 b | Aacactgcggccaacttact |
| PE attL loxP t | ctacgcggtgccatcgtt tgcgcaacgttgttgccatt |
| PE attL loxP b | acggcgtcagcggtttaa aacactgcggccaacttact |
| PE LR loxP t | tcgcgtcctgctgattctg tgcgcaacgttgttgccatt |
| PE LR loxP b | aaagcgatgcggtaggtgtag aacactgcggccaacttact |
| PE malF b | gttaaaaagacagtgatcag aacactgcggccaacttact |
| PE 5kR b | aaatgcaacgccagaaccct aacactgcggccaacttact |
| PE 9KR b | ttagtacaacggtgacgccg aacactgcggccaacttact |
| PE 15KR b | gttaggttgtgaatgagagt aacactgcggccaacttact |
| PE 25KR b | tacagcagataaatgttcta aacactgcggccaacttact |
| PE 37KR b | agcgactgcatgaagattat aacactgcggccaacttact |
| PE 37kL t | aaggtctgccggtaaccgaa tgcgcaacgttgttgccatt |
| PE 5kL t | atgggaacggcagatttgtg tgcgcaacgttgttgccatt |
| PE 5kL b | acatagacatcgttctgcac aacactgcggccaacttact |
| PE 10kL t | gcaccccctcaggtgttatc tgcgcaacgttgttgccatt |
| PE 10kL b | tagctgaactactgaccgcc aacactgcggccaacttact |
| PE 25kL t | gcaacaacaaccgttgctga tgcgcaacgttgttgccatt |
| PE 25kL b | cttatccagcctacgttcgg aacactgcggccaacttact |
| PE λ L t | gggatgcaaaatagtgttga tgcgcaacgttgttgccatt |
| PE λ L b | tagcccggagcaacctgtga aacactgcggccaacttact |
| PE λ R b | tgatacccgcgtattaccgc aacactgcggccaacttact |
| 17 L Top FR | tgctgattctgccctacgtgcgcaacgttgttgccatt |
| 17 L Bot RV | cgccttcaaaagcgatgcaacactgcggccaacttact |
| 17 LR Bot RV | tcccgattcgcagcgcataacactgcggccaacttact |

| **3C Crosslinking primers** | |
| --- | --- |
| **Name** | **Sequence** |
| PstI IN FR | tcgtgaggcactgtggagcaaatg |
| PstI IN RV | gctgaaatgcccgtaattttgcaggc |
| EcoRI OUT FR | gaatgttgctgtcgatgacag |
| EcoRI OUT RV | cgaaagagataaatagttaagagaaggc |
| **RACE PCR primers** | |
| **Name** | **Sequence** |
| Pe* GSP1 | accgtgagccgtcatggctgcaaggcga |
| Pe* GSP2 | gcggccctttacggcgtggtgcgtcat |
| Pe* GSP3 | cgggccgcttcggttttgcccacaccag |
| Pe GSP4 | aatcatgggcttccagcgtggggcggg |
| Pe GSP5 | caccagccgatgttttcggcaaaccagg |
